# Development of a novel splice array platform and its application in the identification of alternative splice variants in lung cancer

**DOI:** 10.1186/1471-2164-11-352

**Published:** 2010-06-03

**Authors:** Ruben Pio, David Blanco, Maria Jose Pajares, Elena Aibar, Olga Durany, Teresa Ezponda, Jackeline Agorreta, Javier Gomez-Roman, Miguel Angel Anton, Angel Rubio, Maria D Lozano, Jose M López-Picazo, Francesc Subirada, Tamara Maes, Luis M Montuenga

**Affiliations:** 1 Division of Oncology, Center for Applied Medical Research, Pamplona, Spain; 2 Department of Biochemistry, School of Medicine, University of Navarra, Pamplona, Spain; 3 Department of Histology and Pathology, School of Medicine, University of Navarra, Pamplona, Spain; 4 Oryzon Genomics, Scientific Parc University of Barcelona, Barcelona, Spain; 5 Department of Pathology, Marques de Valdecilla University Hospital, School of Medicine, University of Cantabria, Santander, Spain; 6 CEIT and TECNUN, University of Navarra, San Sebastian, Spain; 7 Department of Pathology, Clínica Universidad de Navarra, Pamplona, Spain; 8 Department of Oncology, Clínica Universidad de Navarra, Pamplona, Spain

## Abstract

**Background:**

Microarrays strategies, which allow for the characterization of thousands of alternative splice forms in a single test, can be applied to identify differential alternative splicing events. In this study, a novel splice array approach was developed, including the design of a high-density oligonucleotide array, a labeling procedure, and an algorithm to identify splice events.

**Results:**

The array consisted of exon probes and thermodynamically balanced junction probes. Suboptimal probes were tagged and considered in the final analysis. An unbiased labeling protocol was developed using random primers. The algorithm used to distinguish changes in expression from changes in splicing was calibrated using internal non-spliced control sequences. The performance of this splice array was validated with artificial constructs for *CDC6*, *VEGF*, and *PCBP4 *isoforms. The platform was then applied to the analysis of differential splice forms in lung cancer samples compared to matched normal lung tissue. Overexpression of splice isoforms was identified for genes encoding *CEACAM1*, *FHL-1*, *MLPH*, and *SUSD2. *None of these splicing isoforms had been previously associated with lung cancer.

**Conclusions:**

This methodology enables the detection of alternative splicing events in complex biological samples, providing a powerful tool to identify novel diagnostic and prognostic biomarkers for cancer and other pathologies.

## Background

Alternative splicing of pre-mRNA is a post-transcriptional modification essential for the regulation of gene expression and function. Through alternative splicing, multiple transcripts are produced from a single mRNA precursor, widely expanding proteome diversity. Deep sequencing applied to diverse human tissues and epithelial cell lines has recently revealed that more than 90% of human genes undergo alternative splicing [1]. A global analysis of alternative splicing in the human transcriptome suggested that exon skipping is the most prevalent form of alternative splicing [2]. Alternative splicing is a tightly regulated process influenced by cell type, developmental stage, external conditions, etc; however, it is also associated with multiple disease conditions, including cancer [3]. For example, cancer-related aberrantly spliced variants have been shown to be actively involved in the initiation and/or progression of some types of cancer [4]. Splicing alterations are the consequence of splice-site mutations, deregulation of splicing regulatory factors, or both [5]. Tumor-specific variations in splicing may generate new epitopes that can serve as a starting point for immune therapy or targeted delivery, as well as for the development of new diagnostic or prognostic tools [6]. Thus, the identification and molecular characterization of alternative splicing variants associated with cancer is currently a very active area of research [7]. In recent years, powerful techniques for genome-wide identification and analysis of alternative splicing isoforms have been developed. These large-scale high-throughput analytical methods have been applied to the identification of differential splicing events in cancer tissues [8]. Exon microarrays, which contain both known and predicted exons, have been recently used for this purpose [9-13]. However, since they are not specifically designed to examine alternative splicing, they fail to detect events such as the alternative use of 5' or 3' splice sites, intron retention, or the insertion of cryptic exons. Other splicing-specific microarrays have been developed to cover most alternative splicing events. These arrays contain oligonucleotide probes that span exon-exon junctions, and probes positioned within exons to determine individual exon levels and overall transcript expression. The use of splice-junction oligonucleotides to analyze splice events was proposed as early as 1986, when Morgan and Ward used them to identify differential splice forms of minute virus in mice cDNA [14]. In 1996, Lockhart et al. reported one of the first genome-wide microarray studies and suggested the potential of microarrays for the analysis of alternative splicing [15], but it was not until 2002 that Clark et al. developed the first microarray containing splice-junction oligonucleotides to analyze splice events in yeast [16]. In 2003, Johnson et al. used microarrays containing oligonucleotide probes complementary to exon-exon junction sequences to discover new alternative splice variants in human tissues [17,18]. Also in 2003, Wang et al. designed an algorithm that aimed to deconvolute the absolute concentrations of each alternative transcript present in a complex mixture starting from the hybridization intensities detected on splice chips [19]. A new algorithm, called SPACE, has recently been developed for estimating the number of different splicing isoforms (known and unknown), and determining their structures and relative concentrations [20].

Nonetheless, currently available splice arrays still have many limitations, mainly due to problems in the design of the array, the labeling protocol, and data analysis. The development of robust and efficient splice microarrays and data-analysis methods will facilitate progress in the diagnosis, prognosis, and therapy of cancer and other pathologies. In the present work, we describe a novel comprehensive methodology for high-throughput profiling of alternative splicing in complex biological samples. In this methodology, processing of results is based on the array specific design, which is original and thought specifically for alternative splicing-discovery. The strategy consists of optimization of probe design, development of an unbiased amplification protocol that avoids inappropriate transcript coverage due to 3'-biased labeling, and implementation of detailed data processing. Oligonucleotides for the splice array were designed using the Tethys module (Oryzon Genomics, Barcelona, Spain), an inhouse oligo design program, complemented with a new splice-analysis specific module (AltTethys). The algorithm targets the best possible oligonucleotide for each sequence, rather than imposing a strict oligonucleotide quality cutoff. A new labeling protocol was developed to ensure optimal all-length transcript coverage and lineal amplification, working with small amounts of human material. To analyze the data, we developed a novel algorithm for the analysis of two-color arrays that allows for a statistically robust identification of candidate spliced genes in absence of a prior hypothesis about the contributing isoforms. We have applied this technology to the identification of lung cancer-associated splicing variants. Lung cancer is a devastating disease with few therapeutic options or suitable molecular biomarkers for early diagnosis. The results obtained in this study validated the utility of the platform, allowing the identification of new cancer-associated splicing variants with potential utility in the management of lung cancer.

## Results

### Development and validation of the unbiased labeling protocol

The basis for the reliable detection of splice forms in a microarray format is a high-quality, non-biased labeling procedure. Different labeling protocols have been applied in splicing analyses: (a) Castle et al. developed a PCR + T7 amplification-based protocol [17]; (b) a commercial kit is available from Stratagene (Fairplay II) to produce a labeled first-strand cDNA using random hexamers which works well if a sufficient amount of starting material is available; (c) some authors have applied standard 3'-biased RNA labeling protocols, ignoring the loss of 5' events.

**Figure 1:**
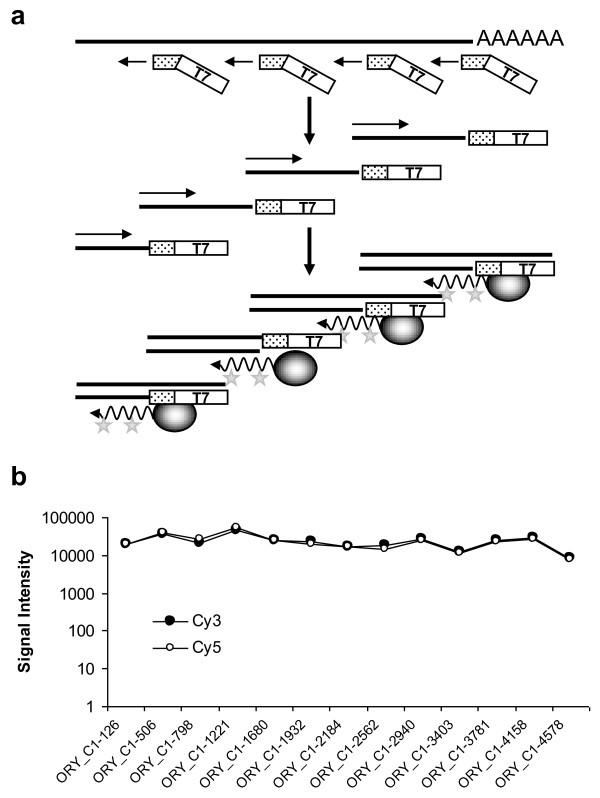
**Label protocol and control genes**. (**a**) Scheme of the labeling protocol, which consisted of the following steps: (I) extraction of polyA + RNA, (II) first-strand cDNA synthesis employing a chimeric primer including the T7 promoter sequences and a stretch of random bases, (III) second-strand cDNA synthesis, (IV) *in vitro *transcription using the T7 RNA polymerase in the presence of a labeled ribonucleotide (Cy3- or Cy5-CTP). (**b**) Probe intensities for the yeast control gene fragment YML059C, demonstrating no 3'- or 5'-biased labeling.

Considering the valuable and limited starting material of human clinical samples (total RNA amounts were < 100 ng), as well as ours and others previous experience in expression microarray studies with cRNA lineal-amplified material by *in vitro *transcription [21], we developed a novel protocol for the detection of alternative splice events consisting of the following steps (Figure 1a): (a) extraction of polyA + RNA; (b) first-strand cDNA synthesis employing a chimeric primer including the T7 promoter sequences and a stretch of 6 random bases starting from 50 ng of polyA + RNA; (c) second-strand cDNA synthesis; and (d) *in vitro *transcription using the T7 RNA polymerase in the presence of a labeled ribonucleotide (Cy3- or Cy5-CTP). For a first validation of performance, three different yeast sequences were tested, ranging between 4500 and 7000 bp to cover any outlier isoform length. PCR-amplified yeast control sequences and the corresponding high-quality yeast control oligonucleotides were used on the microarray surface to assess whether a bias with respect to oligonucleotide position within the transcript had occurred. While some variations in signal intensity of the different oligonucleotides were identified, there was no evidence of 3'- or 5'-end bias (Figure 1b). The same controls were included later in the lung array design. All human samples (both from normal and tumor tissues) were spiked-in with a mix of the three yeast controls to monitor unbiased 3'-5' transcripts coverage and labeling. The yeast control spike-in mix was prepared in different proportions to check significant signals throughout the dynamic range of the microarray experiments. The quantity of the yeast spike-in mix was optimized to avoid interference with the correct amplification of the human samples.

### Performance of the array in the evaluation of artificial splicing forms

In the next step, the hybridization behavior of high-quality oligonucleotides for the yeast controls spiked into the mixture was evaluated and then compared with that of their corresponding thermodynamical half-oligonucleotides. Half-oligonucleotides contain the thermodynamic half of the total control oligonucleotides (located on either the 5' or the 3' end) complemented to the size of the total oligonucleotide with a sequence not expected to hybridize with the yeast control sequence. The half-oligonucleotides thus represent an event in which the DNA on one end of the junction is 100% joined to a different DNA sequence. The use of half-oligonucleotides caused a sharp decrease in signal intensity compared with the complete oligonucleotides (Additional file 1: Figure S1), illustrating that the hybridization temperature and washing conditions were adequate. Nevertheless, most signals did not drop to zero and there was considerable variation in the relative signal intensities of both half-oligonucleotides, indicating that different splice forms can still contribute to the signal of oligonucleotides containing only half of the target sequence of a given transcript.

**Figure 2:**
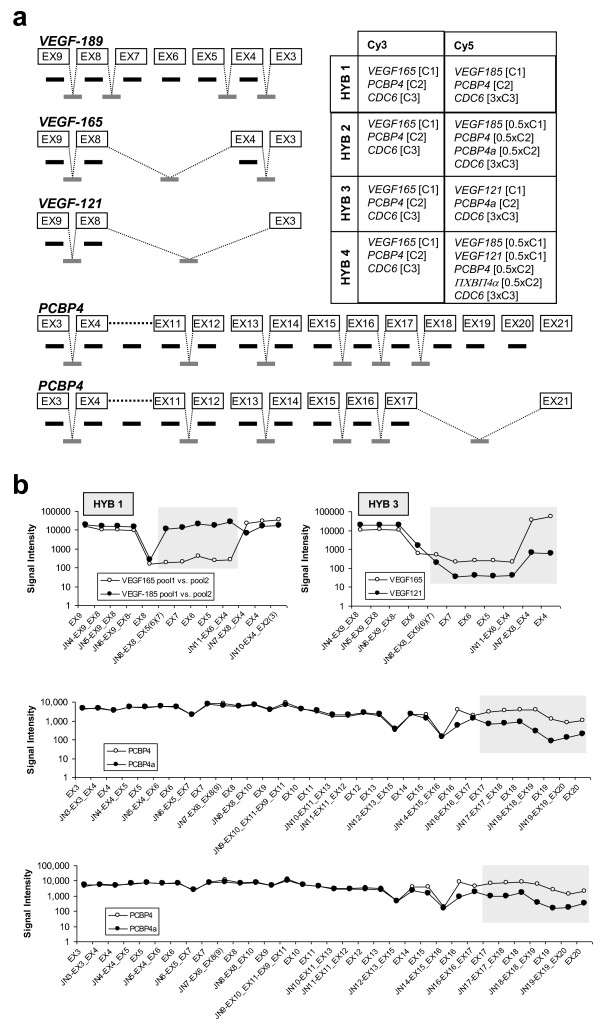
**VEGF and PCBP4 isoforms in the pilot experiment**. (**a**) Simplified organization of *VEGF *and *PCBP4*, showing the included exon (black) and junction (gray) probes, and the hybridization scheme used to evaluate the artificial splicing forms. (**b**) Detection of *VEGF *and *PCBP4 *isoforms in the pilot experiment. Probes with differences in signal intensities are indicated by shadowed boxes.

Subsequently, we evaluated the behavior of our splice array in the detection of aRNA from artificial constructs of *VEGF*, *PCBP4*, and *CDC6 *genes. Three different isoforms were used for *VEGF *(*VEGF*_121_, *VEGF*_165_, and *VEGF*_185_), two for *PCBP4 *(*PCBP4 *and *PCBP4a*), and a single isoform for *CDC6*. Different isoforms were compared at different concentrations, and their respective signal intensities on the pilot array were analyzed. A simplified genomic organization of the *VEGF *and *PCBP4 *exons, as well as the hybridization scheme, is shown in Figure 2a, and the different signal intensities for the relevant probes in Figure 2b. The results confirmed the inherent capacities of the probes to differentiate between different isoforms. While these results were extremely promising, it should be noted that they were obtained with artificial gene fragments present at relatively high concentrations and in an experimental background of reduced complexity (and thus with a reduced potential for cross-hybridization).

### Identification of alternatively spliced genes in lung cancer and selection of genes for validation

The positive results obtained in the pilot array using spiked-in isoforms of individual genes encouraged us to develop a splice array to identify differential splicing variants in complex biological samples, specifically, in clinical samples from patients with lung cancer. Based on gene-expression databases [22,23], 7,958 genes expressed in normal and tumor lung tissue were selected and used to design a splice array. The array contained 115,318 exon probes and 105,141 junction probes for the selected genes, control probes for the yeast YML059C, YOR328, and YIL129C transcripts to monitor the labeling procedure, as well as control probes for maize transcripts (Zm48, Exp and Xet), employed in standard gene expression analyses by Oryzon (both positive and negative controls at different concentrations).

Differential splicing in the 7,958 genes was then assessed with the splice array, by hybridization of 20 pairs of Cy5 labeled tumor and Cy3 labeled normal tissue samples (T_Cy5 _vs N_Cy3_) prepared from 20 non-small cell lung cancer (NSCLC) patients (Additional file 2: Table S1), as well as three self to self comparisons (N_Cy5 _vs N_Cy3_). The array data from this study have been submitted to Gene Expression Omnibus http://www.ncbi.nlm.nih.gov/geo under accession no. GSE18346. The results were analyzed with AltPolyphemus (Additional file 2: Supplementary Methods), which allowed intensity changes of all the oligonucleotides for a given gene to be analyzed with respect to whether these changes reflected gene expression changes or isoform changes. Essentially, after pre-processing (data filtering and normalization) the algorithm first estimates the experimental variability of the microarray analysis platform using the data of the standard deviation of the gene probe data on the replicates of the self to self array.

To identify differences in expression level or splice forms, "change" is first assessed in the self to self hybridization. Any change that is not clearly greater than the inherent variability of the measurement system is considered "no-change". In the self to self hybridization the standard deviation (σ_s,g_) can be calculated from the total gene probe dataset and correlated to the standard deviation of the control probe dataset (σ_s,g _= CF·σ_c_). The data spreading for the total gene probe set was always a little higher than that for the control probes (Additional file 2: Supplementary Methods), which means that the control probes slightly underestimate the experimental variation. In tumor vs normal tissue experiments, it is not possible to measure directly the standard deviation for the no-change situation for the total gene probe data set (as different samples are compared), but the standard deviation on the control dataset (σ_c_) can be measured and the standard deviation for the no-change situation for the total gene probe dataset for the tumor vs normal array can be estimated (σ*_s,g _= CF·σ_c_). Robust change can then be defined as change below or above the threshold TH = ± 3σ*_s,g_, although more stringent cut-off can be applied if desired. Once robust change is defined, the algorithm examines whether the ratios of the signal in the Cy5 and Cy3 channels for the exon and junction probes for a given gene (G) fall within the variability of the experiment (reflecting genes with regular differential gene expression) or outside of that variability (potential splice form variation); i.e. below or above μ_G _± 3σ*_s,g_, μ_G _being the mean value for the ratios of the signal in the Cy5 and Cy3 channels for all the oligos for gene G. Note that μ_G _for a gene with differential expression will be clearly over the threshold for the detection of change (the individual probes are differentially expressed) but the variation among the different probes will be below that threshold (Additional file 1: Figure S2c). For a gene to be selected as a candidate for differential splicing, at least one probe has to fall outside of the limits of the marked threshold. The algorithm considers two hypotheses: the observed hybridization can be explained by a differential mixture of isoforms or by a whole gene expression change, and calculates the error of both hypotheses. If there is a possible isoform change, the algorithm establishes the "Form Change", an arbitrary and empirically defined figure to relatively rank the candidates. Figure S3 in Additional file 1 shows a typical output from the AltPolyphemus software for a candidate gene susceptible to alternative splicing in lung cancer.

AltPolyphemus can identify splice candidates on data from individual tumor vs. normal tissue comparisons or on data from replicate analysis; i.e. on the mean change for every probe in a given dataset. In performing the process on data from a biological replica analysis on a set of tumor vs. normal tissue comparisons, the aim is not only to identify individual splice variants but rather to identify those events that, because of their prevalence, appear to be more relevant. A strict biological replicate analysis was performed on the entire lung cancer set in which: (a) all values for a given oligonucleotide were considered (no outlier elimination of samples was allowed, as this could distort our impression of prevalence of events); (b) the signal cut-off for the oligonucleotide detection was set at the mean signal + 3σ*_s,g _of a negative control and the Agilent background control oligonucleotides, thus selecting for genes expressed clearly above the signal detection level; (c) the fold change cut-off to make the selection was set at the mean fold change of the total array data set ± 3σ*_s,g_, selecting the most relevant changes. Internal assessment of the performance of the algorithm with three self-to-self hybridizations was done for experimental variability. Then AltPolyphemus analyzed the average value from the biological replicate analysis and generated a list of genes ranked by the value of their "form change". This analysis generated 260 potential candidates. Table 1 shows the top 10 genes identified in the analysis.

**Table 1:** Genes with potential splice variants differentially expressed between normal lung and lung cancer tissues, as determined by the splice array

**Ensembl No**.	Gene	Name
ENSG00000123838	*C4BPA*	C4b-binding protein alpha chain
ENSG00000102854	*MSLN*	Mesothelin
ENSG00000164741	*DLC1*	Rho GTPase-activating protein 7
ENSG00000099994	*SUSD2*	Sushi domain-containing protein 2
ENSG00000117399	*CDC20*	Cell division cycle protein 20 homolog
ENSG00000022267	*FHL1*	Four and a half LIM domains protein 1
ENSG00000115648	*MLPH*	Melanophilin
ENSG00000007402	*CACNA2D2*	Voltage-dependent calcium channel subunit alpha-2/delta-2
ENSG00000079385	*CEACAM1*	Carcinoembryonic antigen-related cell adhesion molecule 1
ENSG00000042429	*CRSP6*	Mediator of RNA polymerase II transcription subunit 17

### Validation of splice variants differentially expressed in lung cancer

Differences in alternative splicing between primary NSCLC tissue and normal lung tissue in the ten selected genes were validated by PCR and sequencing. Validation was performed with samples from a group of patients included in the array (Additional file 2: Table S1) and an independent series of NSCLC patients (Additional file 2: Table S2). *IPO8 *was used as the reference gene [24]. Alterations in alternative splicing were confirmed in 4 out of the 10 genes: *CEACAM1*, *FHL1*, *MLPH*, and *SUSD2*.

#### CEACAM1

**Figure 3:**
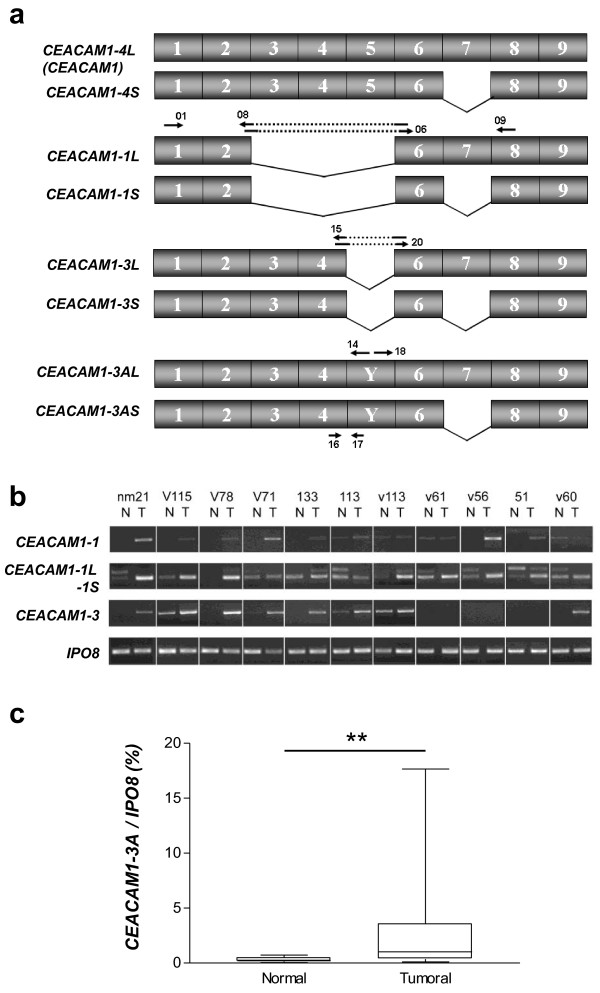
**CEACAM1-1 splicing isoforms in patients with lung cancer**. (**a**) *CEACAM1-1 *splicing isoforms. The location of the primers used in the study is also indicated. (**b**) PCR amplifications of different *CEACAM1 *splice forms in 11 normal lung tissues and paired tumors from patients included in the splice array. *CEACAM1-1 *was amplified with primers 01/08; the L- or S-forms in *CEACAM1-1 *were evaluated with primers 06/09; and *CEACAM1-3 *was determined with primers 01/15. *IPO8 *expression served as the control. Additional PCRs were done but are not shown in the figure: *CEACAM1-3A *was detected with primers 01/14, and L- and S- forms of *CEACAM1-3 *and *CEACAM1-3A *were discriminated using primers 20/09 and 18/09, respectively. (**c**) *CEACAM1-3A *was evaluated by real-time PCR using primers 16-17 in 23 primary NSCLC tumors and their corresponding normal lung samples. ** *p *< 0.01.

Ceacam1 (carcinoembryonic antigen-related cell adhesion molecule 1) is a transmembrane protein involved in intercellular binding and related to several normal and pathological processes [25]. Alternatively spliced forms have been identified for *CEACAM1 *[25]. Our analysis predicted changes in the splicing of this gene around exons 2 and 5. The isoforms generated by alternative usage of these exons are (Figure 3a): *CEACAM1-1 *(lacking exons 3-5), *CEACAM1-3 *(without exon 5), and *CEACAM1-3A *(lacking exon 5 and including an additional exon, hereafter designated as exon Y). To validate the results obtained in the splice array, PCRs were performed with primers specifically designed to identify these three different splice forms (Figure 3a and Additional file 2: Table S3). Samples from primary tumors and their corresponding normal lung tissue were use to evaluate the expression of these isoforms in samples from 24 NSCLC patients, 11 previously included in the splice array (Figure 3b) and 13 from an independent series. In all cases, the identity of the amplified variants was confirmed by sequencing. These results clearly showed that alternative splicing was extensive in lung cancer tissues at the predicted *CEACAM1 *region. *CEACAM1-1 *and CEACAM1-3 were upregulated in tumors compared with their corresponding normal samples (p = 0.029 and p = 0.011, respectively; Additional file 1: Figure S4a). Thus, 92% (22 of 24) of the tumor samples expressed *CEACAM1-1 *whereas only 12 (50%) of the normal lung samples were positive for this isoform. More interestingly, *CEACAM1-1 *expression was upregulated in 18 (75%) of the tumors compared with their respective normal-tissue counterparts. Nineteen tumors (79%) and 12 normal lung samples (50%) expressed *CEACAM1-3*, which was upregulated in 17 tumors (71%). The predominance of the *CEACAM1-3A *splice variant in cancer tissues was confirmed by conventional PCR (data not shown) and quantified by real-time PCR in 23 cases (Figure 3c). *CEACAM1-3A *was significantly upregulated in tumors compared with their corresponding normal samples (*p *= 0.002). This isoform was upregulated in 19 tumors (83%), with an increase in expression ranging from 2- to 70-fold (mean ± SD: 14.2 ± 3.3). The alternative use of exon 7 generates *CEACAM1 *isoforms with either a long (L-form) or a short (S-form) cytoplasmic tail. We analyzed whether the upregulated isoforms *CEACAM1-1*, *CEACAM1-3*, and *CEACAM1-3A *were L- or S-forms using primers specifically designed to detect these variants and concluded that primary lung tumors predominantly express the S-forms (Figure 3b and Additional file 1: Figure S4a). Finally, the expression of *CEACAM1-1*, *CEACAM1-3*, and *CEACAM1-3A *was determined in a panel of 23 lung cancer cell lines derived from tumors of all the major histological subtypes (Additional file 2: Table S4), which demonstrated the expression of these three isoforms not only in NSCLC but also in small cell lung cancer (SCLC).

#### FHL1

**Figure 4:**
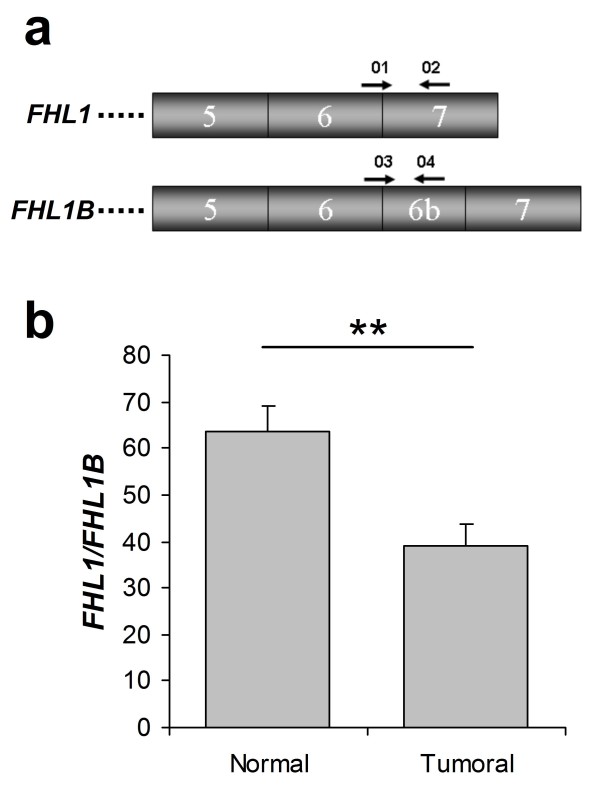
**FHL1 splicing isoforms in patients with lung cancer**. (**a**) Exon structure of the 3' end of *FHL1 *showing alternative use of exon 6b in FHL1B. Primers used to quantify the two isoforms expressed by lung epithelial cells are also shown. (**b**) Ratio between the relative expression of *FHL1 *and *FHL1B *in normal lung tissue and primary NSCLC tissue as determined by real-time PCR. ** *p *< 0.01.

The protein Fhl1 (four and a half LIM domains 1) is mainly involved in skeletal muscle development [26]. Of the alternative splicing variants described for *FHL1 *[27-29], one of the best characterized is *FHL1B *(also called *SLIMMER*), which contains an alternative exon 6, hereafter designated as exon 6b (Figure 4a). Our splice array detected differential changes between normal lung and lung cancer patients in the use of exon 6 and exon 6b. These changes were validated by PCR in 23 samples, 8 samples from patients included in the splice array and 15 additional samples from the independent set. Conventional PCR followed by sequencing revealed the expression of two *FHL1 *isoforms in lung samples: *FHL1 *and *FHL1B *(data not shown). Expression of the two isoforms was specifically quantified by real-time PCR. In agreement with previous reports in other types of cancer [30-32], both were found to be downregulated in lung cancer specimens compared with normal lung tissue (data not shown). Interestingly, a significant change in the ratio between *FHL1 *and *FHL1B *was also observed (*p *= 0.003; Figure 4b). These data validate the differences found by the splice array and suggest that downregulation of *FHL1 *in lung tumors differentially affects one of the two expressed splice forms.

#### MLPH

**Figure 5:**
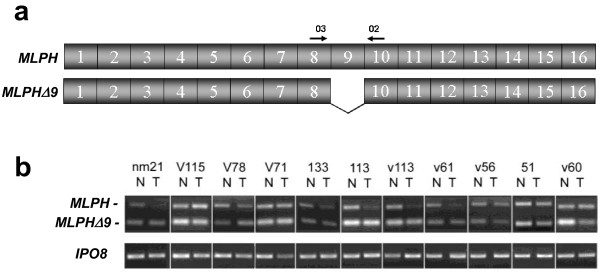
**MLPH splicing isoforms in patients with lung cancer**. (**a**) Exon structure of *MLPH *and the splice variant in which exon 9 is skipped. Primers used for amplification of both isoforms are also indicated. (**b**) PCR amplification of both isoforms in samples from 11 NSCLC patients included in the splice array.

Mlph (melanophilin or Slac2-a) is a protein involved in the transport of melanosomes [33]. The *MLPH *gene contains 16 exons and encodes a protein of 600 residues. Results from our splice array suggested changes in splicing around exon 9, and database analysis indicated the alternative use of this exon in different isoforms (Figure 5a). This alternative isoform generates a protein 28 amino acids shorter than the normal protein. To validate the alternative splicing change, 11 samples from patients included in the splice array were analyzed (Figure 5b). A specific downregulation of the isoform containing exon 9 was identified in 8 out of the 11 cases studied (73%), confirming the results obtained by the splice array (p = 0.036; Additional file 1: Figure S4b).

#### SUSD2

**Figure 6:**
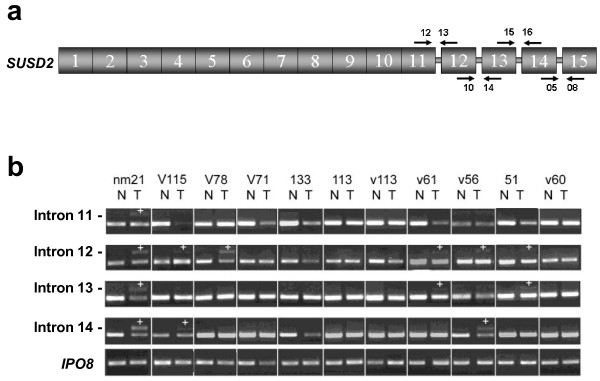
**SUSD2 splicing isoforms in patients with lung cancer**. (**a**) Exon structure of *SUSD2*. The position of the primers used to study the exonization of introns 11, 12, 13 and 14 is shown. (**b**) Determination of the presence of intron retention in samples from 11 NSCLC patients included in the splice array. The lower bands in each gel represent cDNA without exonization; the upper bands correspond to the retention of introns (the presence of an amplified band is indicated by "+"). *IPO8 *expression served as the control.

Susd2 (sushi domain-containing protein 2) is a recently identified single-pass type I membrane protein [34]. Only one *SUSD2 *mRNA variant has been described to date (Figure 6a); however, splice array data suggested lung cancer-associated changes close to the 5' and 3' ends of the mRNA. To validate these results, normal and tumor-bearing lung tissues from 21 cases (11 patients used in the splice array and 10 cases from the independent set) were analyzed, with reduced expression of *SUSD2 *detected in lung cancer tissue compared with normal lung. Regarding changes in alternative splicing, alterations in the 3' end but not in the 5' end of the mRNA were found. In particular, frequent intron retention between exons 11 and 15 was observed (Figure 6b). Inclusion of intronic sequences within an mRNA is termed exonization. In our study, exonization of intron 11 was detected in 3 cases (14%), exonization of intron 12 in 8 cases (38%), exonization of intron 13 in 5 cases (24%), and exonization of intron 14 in 4 cases (19%). Exonization was not observed in any of the normal lung samples analyzed. Since in most of the cancer samples with exonization only one or two introns were retained, amplification of contaminating genomic DNA can be ruled out. Nevertheless, to provide additional verification, *IPO8 *and *GAPDH *were amplified with primers that allowed the detection of genomic DNA and cDNA. Bands for *GAPDH *and *IPO8 *cDNA but not for their genomic DNA were obtained (data not shown). Finally, a panel of 23 lung cancer cell lines was analyzed for the presence of introns retained in the mature mRNA (Additional file 2: Table S4). A high frequency of exonization events was confirmed within *SUSD2 *mRNA not only in NSCLC, but in SCLC as well.

## Discussion

This article reports the development of a platform for the analysis of differential alternative splicing in complex biological samples and its application to the discovery of alternative splice forms associated with lung cancer. Microarray-based methods have been described previously for the identification of splicing events in different physiological and pathological conditions [9-13,35-37]. However, in spite of progress in development and interpretation, splice arrays still have many limitations and are far from attaining the level of standardization and robustness achieved with other high-throughput analytical methods, such as expression arrays [38]. These limitations involve several steps of the process, such as the array's design, the labeling protocol, and data analysis.

The detection of alternative splicing using arrays containing only exon probes is based on the idea that a discrete set of exons, some of which are skipped in the event of alternative splicing, constitute the final mRNA. But this view is largely simplified, as exons may be longer or shorter, junctions may form at different positions, and intron sequences between two exons may be retained. In addition, some exons are very small, to the extent that any oligonucleotide designed to detect them would require the inclusion of sequences from flanking exons. Even in the case of splice arrays that contain junction probes, the design of these probes is challenging. Most splice arrays make use of oligonucleotides of constant length or Tm, but they do not consider that the contributions of the two sequences on either side of the junction may be substantially different due to differences in sequence composition. Probe quality is also affected by the strong spatial restrictions on oligonucleotide design required for the analysis of differential splicing. This leads to an inevitable breakdown of the strict thermodynamic and specificity criteria that are usually imposed on the design of an expression microarray. As for the labeling protocols, methods for standard gene-expression analysis are generally based on labeling from the 3' end, followed by detection with 3'-end probes. However, in a splicing analysis, the sequences of the oligonucleotides present in the array need to spread over the complete length of the transcripts, and the quality of the analysis strongly depends on homogeneous labeling of the RNA. If the labeling protocol is inappropriate, hybridization of insufficient material create "absent" values; more importantly, if the intensity of a fraction (but not all) of the probes for a given gene drops below the detection limit, normal gene expression changes will lead to incorrect detections of splice changes. Consequently, the rate of false-positive discovery would be considerable for genes expressed at low levels. Additionally, in a splice array, data processing and the identification of genuine differential splicing events are more complex than in standard gene-expression arrays and require specific analytical algorithms. This is due to the larger variation in thermodynamic conditions and possible cross-hybridization or folding of sub-optimally designed probes. Finally, an additional requirement for the analytical algorithm is the need to distinguish differential splicing from changes in gene expression levels. The methodology described in the present work has addressed all these limitations.

In designing the probes, we applied an oligonucleotide design algorithm that performs an *in silico *thermodynamic simulation of the hybridization procedure. The algorithm targets the best possible oligonucleotide for each sequence, rather than imposing a strict oligonucleotide quality cutoff. Several control oligonucleotides were also designed and included in the array to control labeling and hybridization processes. The proper design and inclusion of control oligonucleotides, as well as appropriate use of the data generated by these controls in data processing, are especially relevant considering that technical variability can be introduced by the addition of steps in the labeling protocol necessary to avoid labeling biases. To analyze the data, we developed new software to interpret the intensity changes of all the oligonucleotides for a given gene and to decide whether they reflected expression changes or isoform changes.

The efficacy of our new methodology and its potential usefulness in a clinical setting were tested in an application designed to identify genes differentially spliced in primary lung tumors. Lung cancer is the leading cause of cancer deaths worldwide [39], with the major form, NSCLC, accounting for about 80% of all lung cancers. In spite of advances in early detection and treatment, overall 5-year survival rates for NSCLC remain at about 15% [40], underlining the need for a better understanding of the molecular pathogenesis of NSCLC. It has been proposed that modifications in the concentration, localization, composition, or activity of RNA-binding proteins acting as splicing regulatory factors induce the splicing alterations characteristic of lung cancer [41]. In this sense, the abnormal expression of heterogeneous nuclear ribonucleoproteins (hnRNP) in NSCLC clinical samples and animal models suggests that tumors develop specific hnRNP profiles [42-44]. This alteration would generate clinically relevant alternative splice forms contributing to lung carcinogenesis. A recent report presented a genome-wide analysis of alternative splicing events in lung adenocarcinoma [13]. In that study, the authors obtained a list of cancer-related candidate genes showing alternative splicing events and implicated in cancer.

In the present study, the presence of differentially expressed splice variants in NSCLC was evaluated using a splice array designed to detect near 8000 genes known to be expressed in lung tissue. Analysis of the splice array data generated a list of candidates, from which 10 genes were selected for validation. Since one of the main purposes for this selection was to validate the quality of the detection process, no biological criteria were considered in the selection of the candidate genes at this point. RT-PCR experiments, followed by sequencing, were used to validate the results from the array, with changes in alternative splicing confirmed in four genes. As expected, the validation success was below the rates obtained in gene expression studies and was comparable to the rates reported in previous splicing studies [13,18]. Regardless of the platform and algorithm used to detect differential splicing, by microarray or other hybridization-based analysis, it is important to realize that the technology is inherently sensitive to a number of errors that can lead to the incorrect identification of alternative splicing. For example, low-level expression can lead to the erroneous identification of splice events, due to the fact that not all oligonucleotides generate the same level of signal, and the signal of low-responsive oligonucleotides can drop below the detection limit thereby generating false "form changes" when the overall expression level differs between Cy3 and Cy5 channels. Cross-hybridization, obviously, is another potential cause of the detection of false "form changes". While cross-hybridization can sometimes be suspected when the higher signal of one oligonucleotide compared to the others cannot be justified by a much higher Tm or a sub-optimal design, it will generally go unnoticed until further detailed analysis is performed. Moreover, there is no guarantee that all possible gene-structure changes are analyzed in the validation process, unless a very extensive validation approach is applied for any gene of interest (which may be hampered by the availability of clinical material). The four genes with lung cancer-associated alternative splice forms newly identified in this study were: *CEACAM1*, *FHL1*, *MLPH*, and *SUSD2*.

Ceacam1 is a CEA-related cell adhesion molecule downregulated in several human cancer types, including prostate, breast, and colorectal cancers [25]. CEACAM1 has been described as a lung tumor marker, and its expression has been associated with the prognosis of lung adenocarcinoma [45-47]. Two major *CEACAM1 *isoforms have been described: a long (L-) form and a short (S-) form, which, respectively, include or exclude exon 7. The exclusion of exon 7 generates a proximal stop codon that translates into a shorter cytoplasmic domain. Tumor cells transfected with *CEACAM1-1L *are less tumorigenic, suggesting that the L-form functions as a tumor suppressor gene [48]. Wang et al. reported that *CEACAM1-4S *is the predominant isoform in NSCLC tissues, whereas in normal lung tissues the main isoform is *CEACAM1-4L *[49]. This splice pattern was recently confirmed [13]. In addition to confirming previous data, our analysis predicted other changes in the splicing of *CEACAM1 *around exons 2 and 5, which were validated by PCR. For the first time, it was demonstrated that lung tumors frequently overexpress three splice isoforms: *CEACAM1-1*, *CEACAM1-3*, and *CEACAM1-3A*. The alternative use of these exons affects different Ig-like structural domains in the extracellular portion of the respective proteins.

The family of four and a half LIM (FHL) proteins, also known as skeletal muscle LIM proteins (SLIM), is characterized by four complete LIM domains preceded by an N-terminal half LIM domain [50]. LIM domains are cysteine-rich, double zinc-finger motifs involved in protein-protein interactions. FHL has been shown to regulate tissue differentiation, proliferation, adhesion, migration, cytoskeletal organization [51,52], and recently, to play a role in carcinogenesis through a TGF-β-like signaling pathway [53]. Four and a half LIM domains 1 (Fhl1 or Slim1) is a member of this family and has likewise been implicated in skeletal muscle development [26] as well as in the pulmonary vascular remodeling underlying pulmonary hypertension [54]. Interestingly, Fhl1 is downregulated in many types of solid malignancies and it exhibits tumor suppressor activity [30-32]. Among the splice variants described for *FHL1*, in our study the expression of two of them, *FHL1 *and *FHL1B*, was identified in lung samples. In agreement with previous reports, clear downregulation in the expression of the *FHL1 *gene was detected in lung cancer specimens. More importantly, we determined that the downregulation of *FHL1 *is significantly higher than that of *FHL1B*. The two proteins are identical over the first three LIM domains but *FHL1B *contains a distinct C-terminus (96 amino acids) with three potential bipartite nuclear localization signals, a putative nuclear export sequence, and a binding motif for the transcription factor RBP-J [27,28]. Whereas *FHL1 *is mainly located at focal adhesions, *FHL1B *is predominantly a nuclear protein and has unique physiological functions, including the regulation of Notch signaling through its association with RBP-J [55]. Notch signaling profoundly influences the regulation of tumor progression, specifically, tumor cell proliferation, differentiation, apoptosis, and angiogenesis [56].

Mlph is a member of the synaptotagmin-like protein family and is involved in the transport of melanosomes [33]. These lysosome-related organelles are specialized in the synthesis and distribution of melanin. Mlph is an essential member of the melanosome trafficking complex, acting as a link between Rab27a and myosin Va [57,58]. It may also be involved in the trafficking of epithelial Na ^+ ^channel in cells of the collecting duct of the kidney [59]. Mlph contains an N-terminal Slp homology domain (SHD) involved in binding to Rab27a, a myosin-binding domain (MBD) in its middle region, and a C-terminal actin-binding domain (ABD). Here, we demonstrated that lung tissue expresses at least two isoforms of *MLPH*, one with and one without exon 9. Skipping of this exon generates a protein 28 amino acids shorter than the normal protein, without affecting any of the three characterized functional domains. In lung tumors, there is specific downregulation of the isoform containing exon 9.

The recently identified Susd2 is a single-pass type I membrane protein with an extracellular portion that contains somatomedin B, AMOP, von Willebrand factor type D, and sushi/CCP/SCR domains [34]. Although its physiological function is still unknown, overexpression of Susd2 is thought to suppress tumorigenicity [34,60]. In agreement with this postulated role for the protein, we observed reduced expression of *SUSD2 *in lung cancer tissues. Interestingly, intron retention was frequently detected between the last exons of the mRNA. Inclusion of intronic sequences within an mRNA is termed exonization. Although this modification is the rarest type of alternative splicing found in normal cells, exonization events in cancer cells are frequent and may be associated with impairments in splicing regulatory factors [61]. The exonization of introns affects the extracellular portion of *SUSD2 *in that the translation of intron 11 introduces a premature stop codon which disrupts the von Willebrand factor type D domain at amino acid 631. Exonization of intron 12 generates a protein whose last 41 amino acids are substituted by 68 new amino acids (38 coded by intron 12 and 30 new amino acids translated as consequence of a frame-shift in the reading frame of exon 13). Translation of intron 13 generates a protein with a new sequence of 23 amino acids from position 781 (without affecting any known functional domain), while retention of intron 14 introduces a premature stop codon, eliminating seven amino acids at the C-terminal end.

## Conclusions

We have developed and tested a novel platform for high-throughput analysis of alternative splicing events in biological samples. The application of this methodology will aid in understanding the functional relevance of splice variants in pathological conditions and facilitate the identification of new biomarkers and targets for therapy. To prove the usefulness of this platform, this methodology was used to identify cancer-associated splice variants in lung cancer. Differentially expressed splice variants of four genes were identified, with potential utility in the diagnosis of lung cancer. Additional work is in progress to analyze the relevance of these newly characterized cancer-associated isoforms as well as to validate additional candidates from data obtained in the splice array.

## Methods

### Clinical samples

Primary tumors and their corresponding normal lung tissues were obtained from patients with non-small cell lung cancer (NSCLC) treated with curative resectional surgery at the Clínica Universidad de Navarra (Pamplona, Spain) or at the Hospital Marqués de Valdecilla (Santander, Spain). None of the patients received chemo- or radiotherapy prior to surgery. The study was approved by the ethics committees of the participating institutions and informed consent was obtained from each patient. Surgically removed samples were immediately frozen in liquid nitrogen and stored at -80°C until use. A portion of each sample was sectioned in a cryostat and mounted onto slides. After fixation, these samples were stained with hematoxylin and eosin, and then carefully examined by two experienced researchers. Samples containing less than 70% tumor cells were discarded. The 42 specimens selected for the study were divided into two groups: one for discovery (*n *= 20; Additional file 2: Table S1) and one for validation (*n *= 22; Additional file 2: Table S2).

### Lung cancer cell lines

All lung cancer cell lines were obtained from the American Type Culture Collection (ATCC), except HCC44, HCC827, EPLC-272H, and HCC15, which were obtained from the German Collection of Microorganisms and Cell Cultures (DSMZ). Cells were grown in RPMI supplemented with 2 mM glutamine, 10% fetal bovine serum (FBS), 100 U/ml penicillin, and 100 μg/ml streptomycin.

### New splice array design

Oligonucleotides for the splice array were designed using Tethys software (Oryzon Genomics, Barcelona, Spain) complemented with a new splice-analysis specific module (AltTethys) generated for the present study. Tethys is an oligonucleotide design algorithm that performs *in silico *thermodynamic simulation of the hybridization procedure previously used in the design of gene expression or genome hybridization arrays. The array design included exon and junction oligonucleotides to detect genes expressed in the human samples, and oligonucleotides for the detection of artificial spiked-in control genes from yeast. The control oligonucleotides served a dual purpose: to control labeling and hybridization, and to provide relevant statistical information for data analysis.

**Figure 7:**
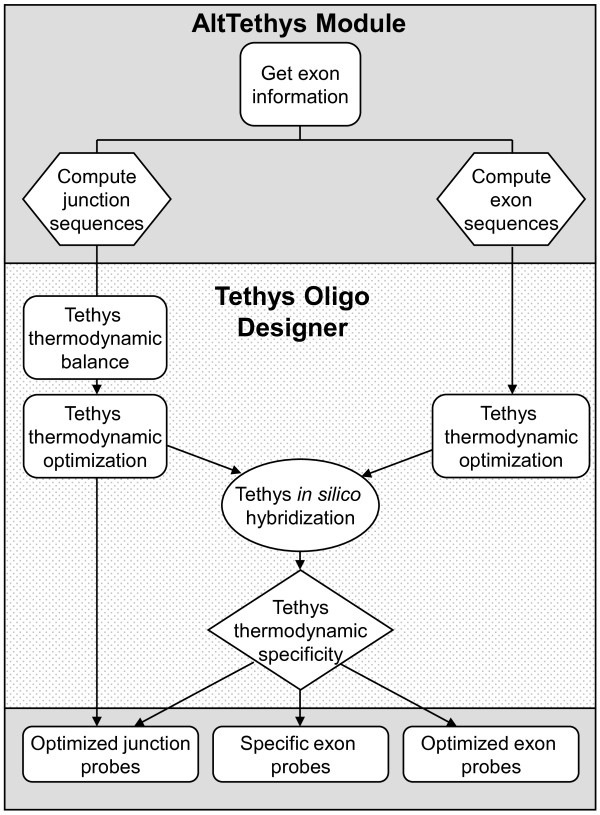
**Flow diagram of splice array oligonucleotide design**. The oligonucleotides were designed using Tethys software (Oryzon Genomics) complemented with a novel splice-analysis module (AltTethys) specifically developed for the present study.

The overall flow of the algorithm used for gene-probe design in the present work is diagrammed in Figure 7. Transcript (exon and junction) information was extracted from Ensembl database (NCBI 36, release 40). A target-sequence database was generated, including all the possible transcripts for a given gene specified in the database. The new AltTethys module located the exons corresponding to each gene and selected the target sequences, which had a target length of 36 bp. If an exon was less than 36 bp long, additional bases were included from the neighboring exons on both sides (Additional file 1: Figure S5). For each transcript, junction target sequences were selected for each known exon-exon junction, including 25 bp at each boundary side and, if an exon was smaller than 25 bp, sequences were added from neighboring exons (Additional file 1: Figure S5). Tethys was then used to design oligonucleotides for the target sequences. Due to the very small degree of freedom resulting from restrictions on oligonucleotide locations, the design targeted the best possible oligonucleotide for each sequence rather than imposing a strict oligonucleotide quality cutoff. However, oligonucleotide quality was considered in the interpretation of the results (see below). Probe synthesis was outsourced to Agilent Technologies.

#### Exon probes

For exon probes, the target Tm was 75°C and the Tm range 70-80°C, with a length modulation of 30-36 bp and a temperature limit of 60°C for cross-hybridization. If no specific exon probe complied with these conditions, the best possible oligonucleotide (minimal cross-hybridization Tm and secondary structure folding Tm) was selected.

#### Junction probes

Junction probes were designed using junction target sequences with a total maximum length of 50 bp. Probe sequences with different lengths but with Tm values as similar as possible for the two sections of the oligonucleotide flanking both sides of the junction were selected. The total length of the junction probes ranged from 25 to 42 bp. Among all possible sequences, the oligonucleotide with a Tm as close as possible to the target temperature of 75°C was selected. Finally, the oligonucleotide was checked for Secondary Structure Folding Tm and Maximum Cross-Hybridization Tm using the Tethys oligonucleotide design backend, taking note of the potential for cross-hybridization.

#### Control probes

An extensive battery of probes was included to control labeling and hybridization processes and to provide relevant statistical information for data analysis: (a) three yeast artificial target sequences were spiked into the hybridization mixture at three different concentration levels but balanced in the Cy3 and Cy5 channels; (b) optimum oligonucleotides (in terms of specificity and thermodynamics) distributed over the length of the target sequences to assess labeling bias; (c) half oligonucleotides (generated by splitting optimum oligonucleotides into two thermodynamically equivalent halves complemented with stretches of AT at the 5' or 3' end) to simulate differential splicing at the splice-donor or splice-acceptor site. These probes were included more than ten times in the array in order to determine intra-array variability.

In addition, a higher number of positive and negative control probes were distributed over the array surface (maize expansin, ZmMYB42, and xyloglucan endo-transglycosylase). These probes were used to assess detection limits and range, to verify spatial homogeneity, and to determine experimental within-array variation.

### Cloning of artificial constructs for VEGF, PCBP4, and CDC6

The performance of the splice array was tested using a pilot array designed to identify different transcripts of three genes: *VEGF*, *PCBP4*, and *CDC6*. Artificial transcripts were generated for three *VEGF *isoforms (*VEGF*_121_, *VEGF*_165_, and *VEGF*_185_) [62], two *PCBP4 *isoforms (*PCBP4 *and *PCBP4a*) [44], and the only known *CDC6 *isoform.

### Preparation of yeast controls

Three yeast sequences of DNA were amplified by PCR from genomic DNA of Saccharomyces cerevisae strain S288C using two chimeric primers, where a primer consisted of a T7 promoter and the gene specific sequence, and the other primer consisted of a tail of 20 timidines and the gene specific sequence. These three genes were: YIL129C (7100 bp), YML059C (4900 bp) and YOR328W (4600 bp). They were amplified by PCR with 32 ng of genomic DNA from yeast and using a combination of two polymerase TaqI:Pfu (20:1). cRNA was generated using an in vitro transcription system (T7 Megascript kit; Ambion) getting the final artificial unique splice forms. The sequences were spiked into the samples prior to labeling.

### RNA extraction and labeling

Total RNA from paired normal and tumor samples from lung tissues was extracted using the RNeasy Extraction Kit (Qiagen) according to the manufacturer's instructions, with minor modifications. RNA quality was assessed using an Agilent Bioanalyzer 2100 and quantified using a Nanodrop ND-1000 spectrophotometer. Samples with an RNA integrity number (RIN) below 7 were excluded from further analysis. PolyA + RNA was extracted using Dynabead magnetic particles. To obtain homogeneous labeling of the RNA across the entire length of the transcript, a novel labeling procedure, described in the Results section, was developed. Fifty nanograms of PolyA + RNA from normal tissue was labeled with Cy3 and the same amount of PolyA + RNA from tumor samples with Cy5. In addition, 50 ng of PolyA + RNA from normal tissue was labeled with Cy5. Prior to labeling, artificial yeast transcipts were spiked into all polyA + samples mixtures. The quality of the labeled samples was verified using the Agilent 2100 Bioanalyzer and sample concentration was determined using the Nanodrop ND-1000 spectrophotometer.

### Array hybridization and data acquisition

Labeled normal tissue cRNA (4.5 μg) was mixed with the same amount of labeled tumor cRNA from the same patient, and equal quantities of Cy3 and Cy5 labeled Xet and Zm42 cRNA controls were spiked in to serve as hybridization controls. The cRNA was mixed with 25 × fragmentation buffer (Agilent) and incubated at 60°C for 30 min to fragment RNA. Afterwards, 250 μl of 2 × hybridization buffer (Agilent) was added to stop the fragmentation reaction and the mixture was hybridized on the array. Slides were incubated for 17 h at 60°C in an Agilent DNA Hybridization Oven (G2545A) with the rotation setting at 4 rpm. A total of twenty Cy3 labeled normal and Cy5 labeled tumor lung cancer samples, were cohybridized pairwise on the splice array as well as three Cy3 labeled normal and Cy5 labeled normal samples. Raw data were acquired using an Agilent DNA Microarray Scanner and Agilent Feature Extraction Software (V.9.1). The general reproducibility of the hybridization platform (labeling procedure, hybridization, and detection) was assessed by means of self to self hybridization; the standard deviation of the fold change of all oligonucleotides was 0.093.

### Data processing

For data processing, a novel algorithm that distinguished between changes in gene expression and splicing variation was developed. The analysis of differential splice isoforms is more complex than the analysis of differential gene expression, due to a higher variation in thermodynamic conditions and possible cross-hybridization and folding of sub-optimally designed oligonucleotides. In addition, compared with regular gene expression analyses, additional variation can be introduced due to the incorporation of extra steps in the labeling protocol. This gives a special importance to the incorporation of spiked-in controls in the array design and their use in data processing. The data processing procedure (which is detailed in Additional file 2: Supplementary Methods) was divided into four steps: data filtering and normalization, probe spot calibration, gene probe statistical analysis, and isoform analysis. Data processing is discussed more extensively in Additional file 2: Supplementary Methods.

### Validation of cancer-associated splice variants by PCR

Results obtained in the splice array were validated by PCR. Two micrograms of RNA from the clinical samples were reverse transcribed. Genomic DNA contamination was controlled in each RNA sample using a reaction mix lacking reverse transcriptase. One microliter of cDNA diluted 1:10 was used for PCR amplification, and the PCR products were electrophoresed in agarose gels. For sequencing, the amplified bands were purified using the Qiagen MinElute PCR Purification Kit and sequenced in an ABI377 sequencer (Perkin-Elmer Applied Biosystems). Real-time PCR was performed using SYBR Green PCR Master Mix (Applied Biosystems) in the Applied Biosystems 7300 Real-Time PCR System. The reactions were carried out according to the manufacturer's instructions. Each sample was analyzed in triplicate. Relative levels of expression were determined by the Ct method using *IPO8 *as the reference [24]. Primers used for validation are shown in Additional file 2: Table S3.

## Abbreviations

NSCLC: non-small cell lung cancer; Ceacam1: carcinoembryonic antigen-related cell adhesion molecule 1; SCLC: small cell lung cancer; Fhl1: four and a half LIM domains 1; Mlph: melanophilin or Slac2-a; Susd2: sushi domain-containing protein 2; hnRNP: heterogeneous nuclear ribonucleoproteins.

## Authors' contributions

RP, OD, TM and LMM conceived and designed the experiments. DB, MJP, TE, JA and MAA performed the lung cancer molecular biology experiments. OD, EA, FS and TM carried out the bioinformatics, platform design and hybridization. RP, DB, EA, OD, TE, JA, MAA, AR, FS, TM and LMM analyzed the data. JGR, MJP, MDL, JMLP and FS contributed reagents/materials/analysis tools. RP, DB, EA, OD, FS, TM and LLM wrote the paper. All authors read and approved the final manuscript.

## Supplementary Material

Additional file 1 **Supplementary Figures S1 to S9**. Click here for file

Additional file 2 **Supplementary Methods and Tables S1 to S4**. Click here for file
