# Supplementary material for: Development of a novel splice array platform and its application in the identification of alternative splice variants in lung cancer
Source: BMC Genomics. 2010 Jun 3;11:352. doi: 10.1186/1471-2164-11-352 (PMC2889901; doi:10.1186/1471-2164-11-352)
Supplement: Additional file 1 — Supplementary Figures S1 to S9. [file 1471-2164-11-352-S1.pdf]

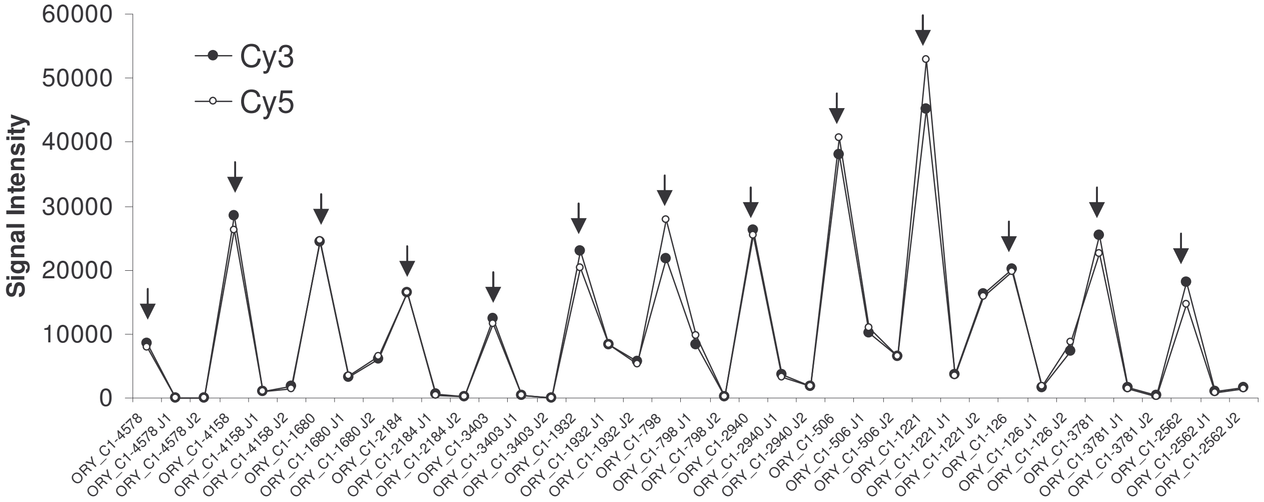

Supplementary Figure S1. Signal intensities of oligonucleotides (arrows) and their 5’ and 3’ half oligonucleotides for a yeast control gene. The used of half-oligonucleotides caused a sharp decrease in signal intensity compared with the complete oligonucleotides, illustrating that the hybridization temperature and washing conditions were adequate.

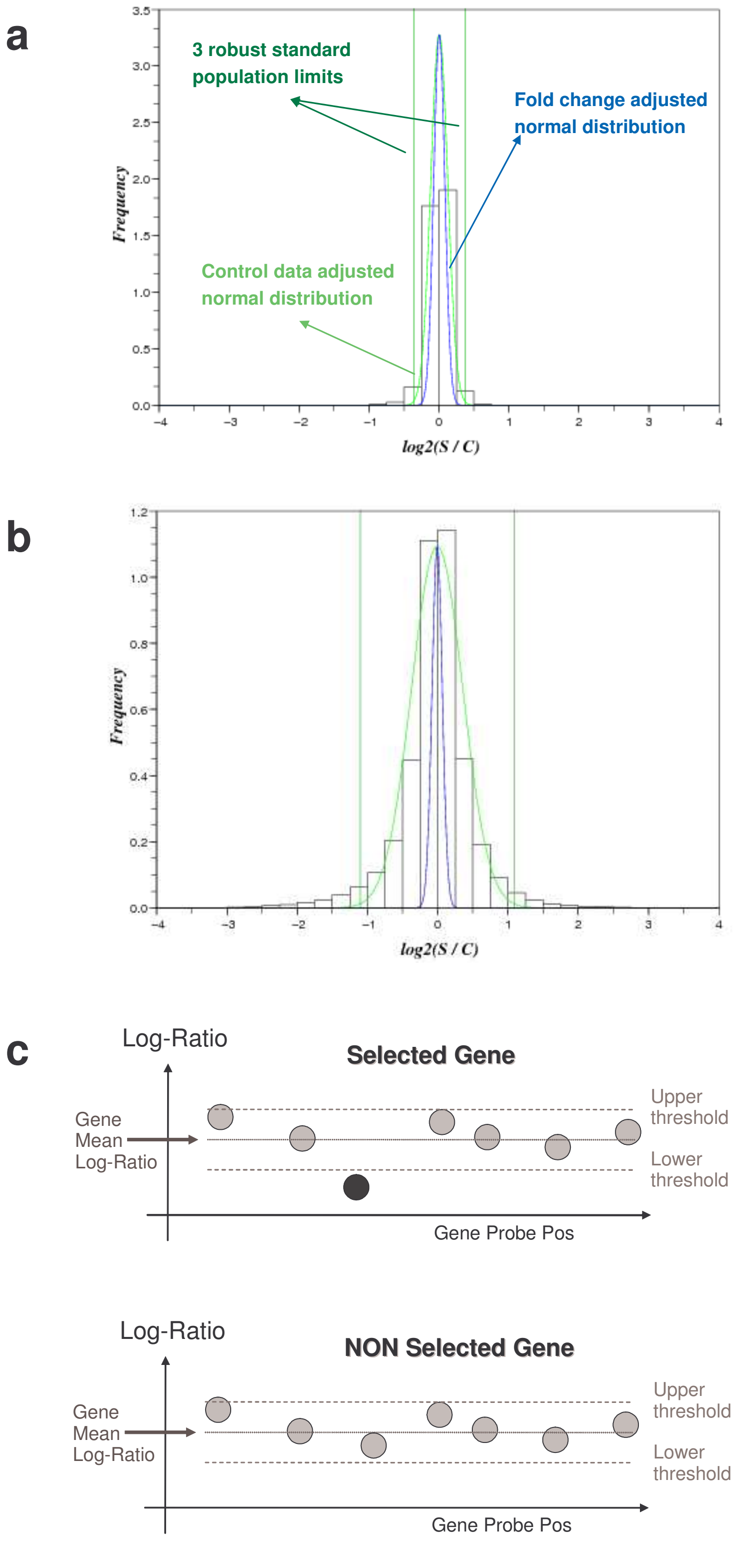

Supplementary Figure S2. (**a**) Histogram of a self to self hybridization. (**b**) Histogram of control (normal tissue) and sample (tumor tissue) dataset fold-change distribution. (**c**) Selection of candidate splice events based on statistical population analysis.

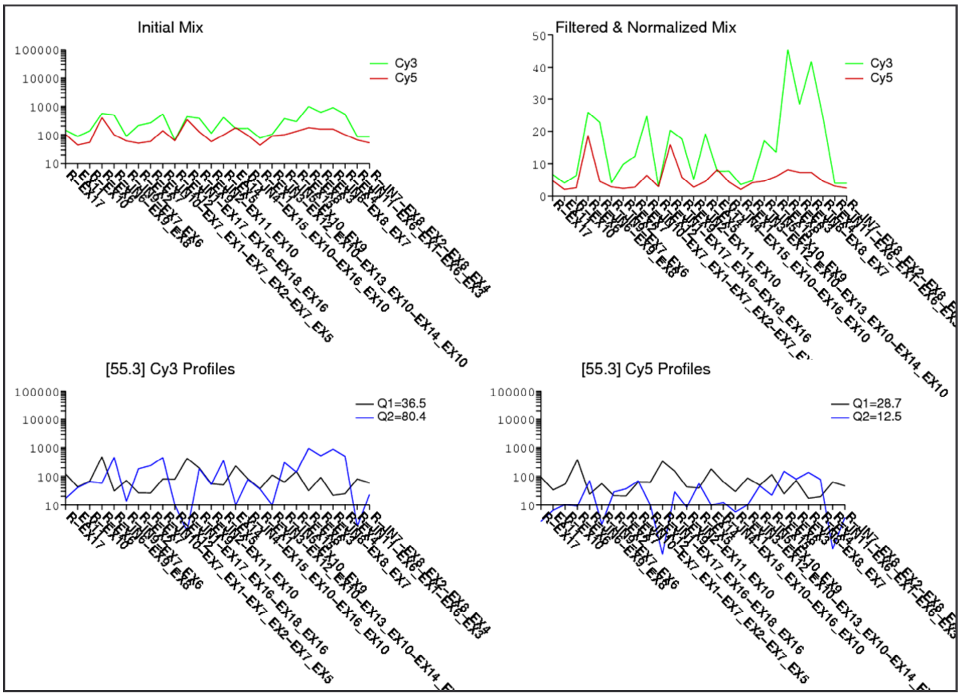

Supplementary Figure S3. Examples of graphs representing the absolute and scaled hybridization intensities (top) and the resolved aggregated patterns (bottom) for the Cy3 and Cy5 channels with their respective Q values. Red curves: tumor tissue; green curves: normal tissue. These graphs correspond to a gene with potential alternatively spliced isoforms.

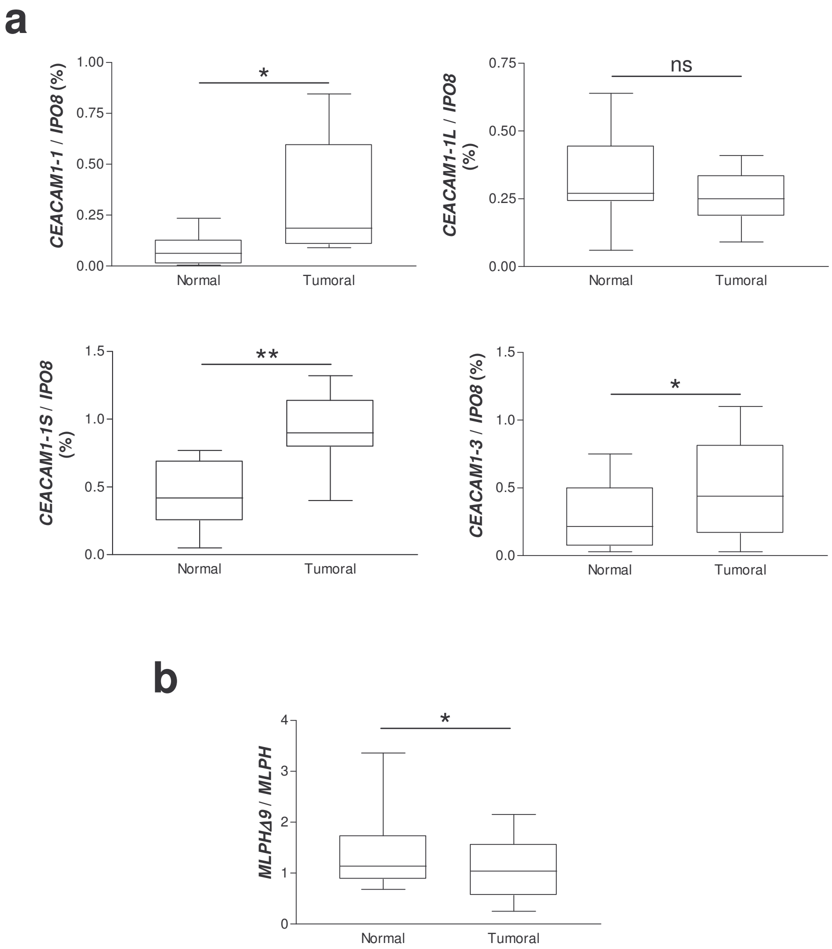

Supplementary Figure S4. Quantification, by densitometry, of PCR amplification products. (**a**) CEACAM1 isoforms (from gel in Figure 3b). (**b**) Ratio MLPHΔ9/MLPH (from gel in Figure 5b).

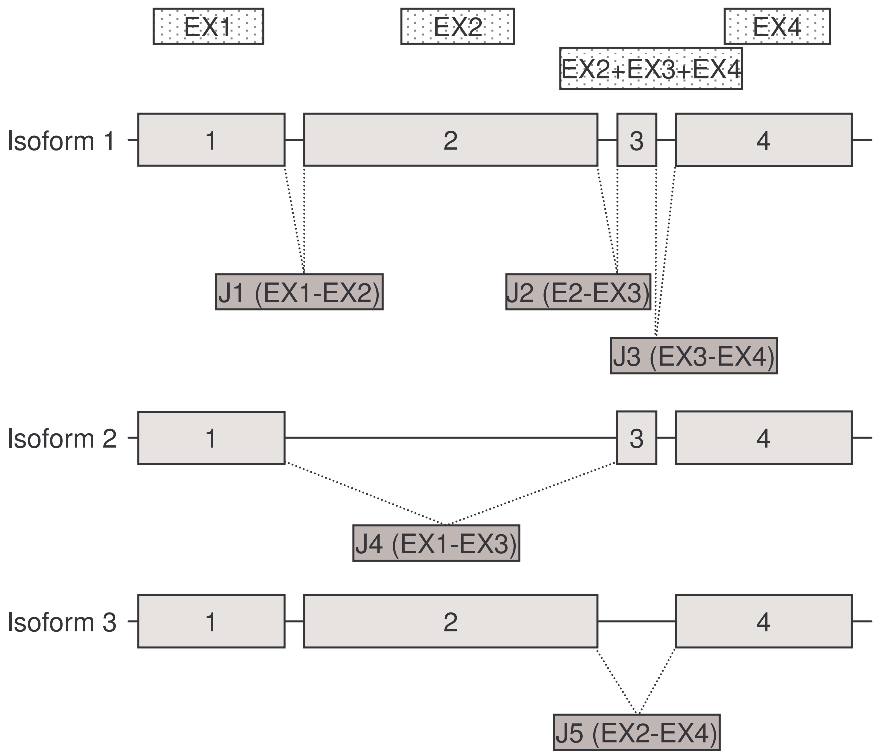

Supplementary Figure S5. Representation of exon and junction target sequence selection

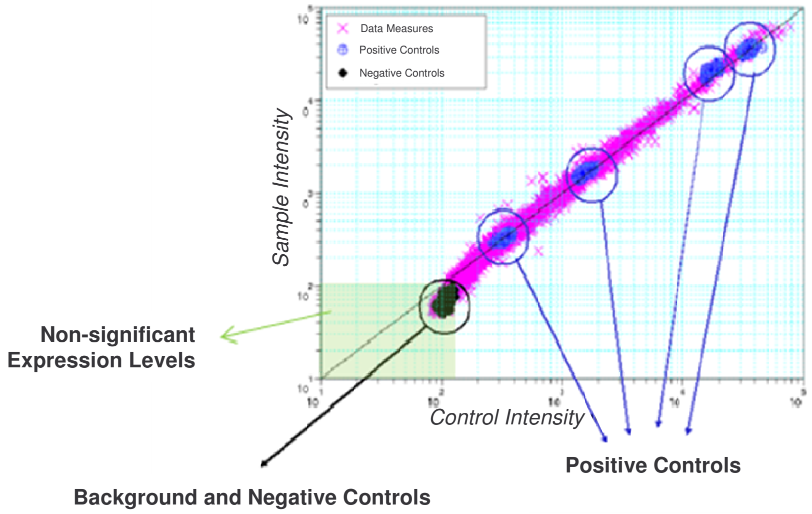

Supplementary Figure S6. Spiked positive controls and the non-spiked (but designed and included in the array) negative controls in a self to self experiment. The analysis of the signal levels for the negative controls plus the Agilent background controls (self fold probes) helped to define a significant signal cut-off in each array.

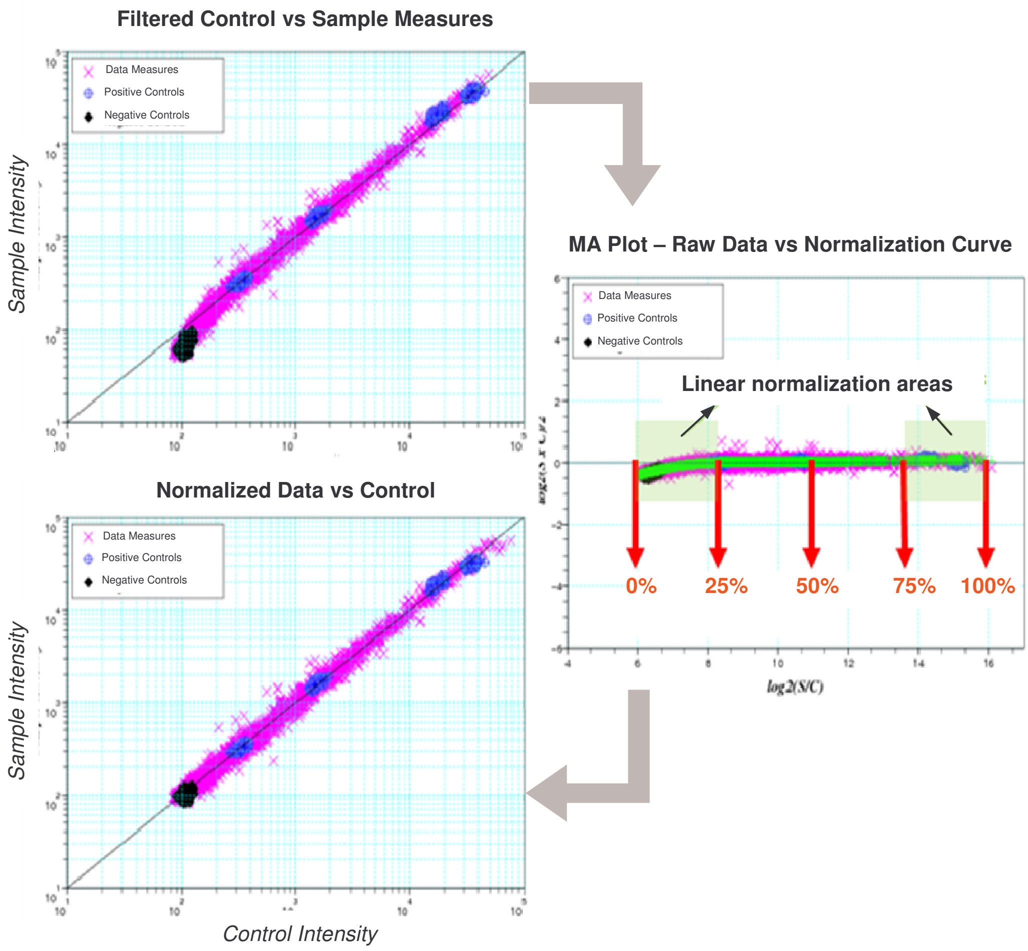

Supplementary Figure S7. Normalization process using the Polyphemus piecewise Q-Splines approach with extremum linear normalization

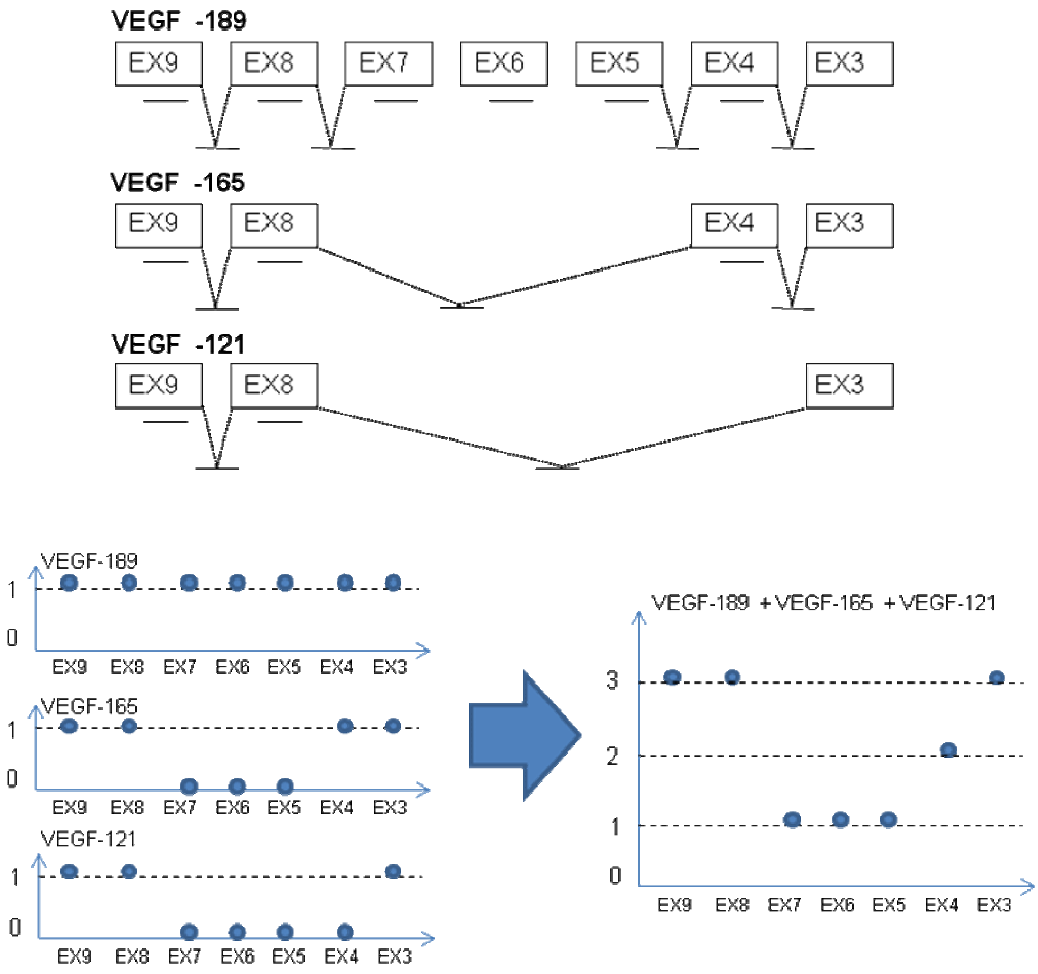

Supplementary Figure S8. This figure illustrates the mixture expected for the VEGF gene, which has three different forms in a single sample. This is the main basis to apply Multivariate Curve Resolution.

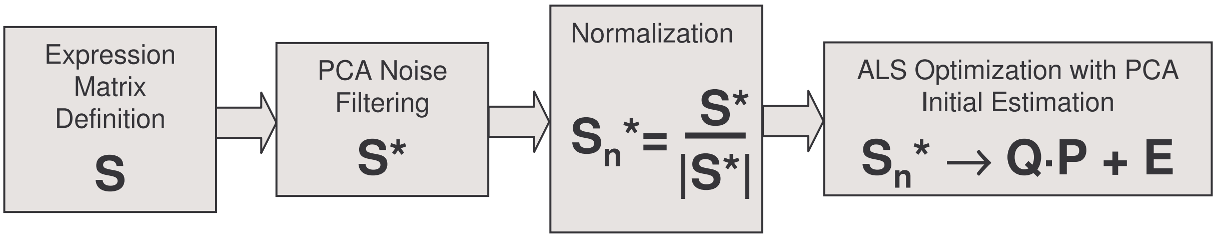

Supplementary Figure S9. Isoform analysis pipeline using MCR-ALS techniques.
