# Supplementary material for: Development of a novel splice array platform and its application in the identification of alternative splice variants in lung cancer
Source: BMC Genomics. 2010 Jun 3;11:352. doi: 10.1186/1471-2164-11-352 (PMC2889901; doi:10.1186/1471-2164-11-352)
Supplement: Additional file 2 — Supplementary Methods and Tables S1 to S4. [file 1471-2164-11-352-S2.pdf]

Supplementary Material and Tables

Supplementary Methods

Choice of hybridization technology

Dual color arrays were chosen for the analysis of splicing in paired tumor/normal tissue samples. In co-hybridization studies, the labeled material from each sample competes with its paired sample on a specific oligonucleotide probe spot, and the relative intensities are representative of the relative contribution of each sample when the hybridization equilibrium is reached. Inherently, co-hybridization eliminates the variation associated with the use of independent arrays, washes, detections and *in silico* comparison of single channel experiments. On the other hand, co-hybridization can introduce a dye bias, which nevertheless can be measured in self to self hybridizations and corrected, if necessary. In our experience, in high quality experiments, the dye bias is small and a correction is hardly ever needed.

Data processing

For data processing, a novel algorithm was developed to distinguish between gene expression changes and splicing variations. The analysis of differential splice forms is more complex than the analysis of regular gene expression, due to a higher variation in thermodynamic conditions and possible cross-hybridization, folding or sub-optimally designed oligonucleotides. In addition, compared with regular gene expression analyses, additional variations can be introduced due to the incorporation of extra steps in the labeling protocol. In this situation, the use of spiked-in controls for data processing is especially important. Data processing was divided into four steps: data filtering and

normalization, probe spot calibration, statistical analysis of gene probes, and isoform analysis.

Data filtering and normalization

The initial steps of data processing were performed as in gene expression arrays described elsewhere (Cerdá et al. 2008). Due to the intrinsic nature of the splice array experiments, we had to develop new processes, very tied to the array design and experimental execution, to overcome a specific problem: a predictable big amount of non-responsive oligonucleotides probes (sub-optimal probes, non-expressed exons, etc) may bias the statistics analysis and distort the normalization at the lower signal level. Therefore, prior to the normalization of the array data, probes that were judged non-responsive were filtered. Specific negative and background control probes were designed and used in all hybridizations (Additional file 2: Figure S6). The signals of these controls in each channel were used to calculate the detection limit as the mean signal value + 3σ for each array. Then, probes with signal intensities below each array's detection limit in all the replicated arrays were filtered out (this process was called Global Filtering).

To normalize the data passed through Global Filtering, MA space was used (Dudoit et al. 2002). M values (vertical axis) are the log-differential expression ratios, and A values (horizontal axis) are the log-intensity of the spot. M and A are calculated from the Cy3 and Cy5 intensity values. The method used was Polyphemus Q-Splines Normalization, in which array data are normalized using an improved version of the non-linear normalization described previously (Workman et al. 2002), with a piecewise approach: using Q-Splines for the inner pieces and linear for the two extremum ones, ensuring a smooth normalization curve (Additional file 2: Figure S7). The data used to make the

normalization did not include the signals derived from positive or negative control probes.

The normalization process is useful to correct deviations of the M values from the statistical assumption that most of the spots have M=0 in MA plots. The method allows the adjustment of all M values to form a cloud centered at M=0 in all intensity ranges. It is important to note that any normalization procedure makes two basic assumptions: the total intensity in both samples is equal, and the data cloud should be centered at M=0 in all intensity ranges. It is also important to keep in mind that normalization cannot correct data saturation or generate data below the original detection level.

The positive and negative hybridization controls were thus helpful to monitor the entire process: to control lower detection limits, signal distribution and saturation, and to control that the final normalization had been performed adequately (the positive spikes should be positioned at M=0 after normalization).

In gene expression studies, intra-array replicate analyses are generally applied at this point. The splice arrays used in this study did not contain triplicate copies for each probe, as replicates for all the probes did not fit on the probe surface at the probe densities used. Additional replicate hybridizations were not performed either, due to a limitation in the amount of material available from the biological samples.

Data processing can be performed on individual arrays, although the absence of intra-array replicates does not support the elimination of bad data points by outlier analysis, which renders the analysis more error prone.

Probe Spot Calibration

The objective is to distinguish intensity changes from random variation with reasonable reliability. For this purpose, both the variability of the intensity of the control probes

within each array and the variability of the intensity of self to self hybridized samples, as assessed by the gene probes, were evaluated. In both cases, the logarithm of the ratio of both channel expression values was statistically analyzed, and the mean and standard deviation for the given log-ratio population was calculated. Typically, the distribution of the control values obtained from the artificial spiked-in controls is a bit narrower than that of the self to self hybridization (Additional file 2: Figure S2a and b).

The two-fold change distribution provides information about the empirically significant change limit, since the threshold for statistically significant changes in probe response cannot be less than the technologically intrinsic variation. Controls are used in all the experiments (self to self comparisons and normal to tumor tissue comparisons) in equal concentration in both channels, thus allowing variations in self to self hybridization to be related to variations in the control. A very simple way to do this is to determine the ratio of both standard deviations, which yields the *Calibration Factor* (CF):

$$CF = \frac{\sigma_{Gene\_\mathrm{Pr}obes}}{\sigma_{Control\_\mathrm{Pr}obes}}$$

First-pass analysis: gene probe statistics

The first step is to decide whether the variation in the logarithm of the ratio of both channel expression values for all the exon and junction probes of a given gene falls within (reflecting genes without or with differential expression) or outside of (potential splicing variation) the variability of the experiment. To differentiate these situations the standard deviation (σ_c) on the control probe was calculated and, using the CF from the calibration, converted in an estimate of the associated gene self to self standard deviation ($\sigma^*_{s,g}$):

$$\sigma^*_{s,g} \approx CF \cdot \sigma_c$$

Subsequently, the threshold was defined as TH = $\mu_G \pm 3\sigma^*_{s,g}$, μ_G being the mean value for the ratios of the signal in the Cy5 and Cy3 channels for all the oligos for a given gene G. Depending on the sensitivity and reliability required, the threshold can be modulated. Genes that contained at least one probe with a log-ratio below or above the threshold were selected for next step in the analysis (Additional file 2: Figure S2c).

Second-pass analysis: isoform quantification

Facing the challenge of doing high throughput screening of clinical samples to search for alternative splice variants, a multidisciplinary integrated approach was required. Array design, sample preparation, labeling, and final data analysis were considered as an interdependent system. Due to the array design, a huge variety of probe-sample interactions was expected: from small sub-optimal junctions to highly cross-hybridized exons. A dual color approach was chosen, because in this technology, based in the co-hybridization of the two compared samples in the same array, at high concentrations the observed fold change is monitoring the biochemical equilibrium of the samples competent for hybridization. This equilibrium is expected to be proportional to the transcript ratio, but the signal level would not be related to the real transcript concentration.

Different algorithms for the quantification of isoforms have been described, including *Wang et al 2003* and *Anton et al 2008*. Both of them use a model assuming a linear relationship between the signal level and the transcript concentration, which is not applicable for the dual color arrays used in this study. Besides, Wang's algorithm explains the signals obtained as a sum of varying amounts of pre-defined transcripts by deconvoluting a signal into a set of contributions of known isoforms, which cannot be applied to predict unknown splicing isoforms. Our objective was different: to reliably

detect that splicing exists in a gene, without trying to determine what the contributing isoforms are. So a new approach was needed to process the data: the AltPolyphemus software.

Given the fact that there is no validated mathematical model to explain the dual color hybridization, a more pragmatic approach was taken to evaluate the expression pattern for a given gene that has different contributors, the spliced transcripts. By analogy, each transcript can be considered a compound and the total gene expression the final mixture; this makes the Multivariate Curve Resolution (MCR) technique, used in chemometrics, a good choice to analyze the splice array data. Multivariate self-modeling curve resolution comprises a group of techniques aimed at recovering the response profiles (spectra, pH profiles, time profiles, elution profiles, etc.) of more than one component in an unresolved and unknown mixture when no or little prior information is available about the nature and composition of those mixtures (de Juan and Tauler 2001; de Juan and Tauler 2003; Tauler 1995; Tauler et al. 1995). MCR has not often been applied to the analysis of regular gene expression data from microarrays (Jaumot et al. 2006; Wentzell et al., 2006), but not to alternative splicing. Translating MCR to the analysis of differential splicing, for each gene a probe signal matrix, *S* (2 colors x n probes), is represented as the product of two lower rank matrices, of *Q* and *P*, which represent the quantification factors and the canonical shape patterns, and a residual error term, *E*. The data matrix is set up based on the assumption that each channel has a linear combination of two or more patterns of the hybridization signals derived from different transcript variants (Additional file 2: Figure S8). The adaptation in matrix form is:

$$S = Q \bullet P + E$$

Where *S* is the expression signal matrix from the hybridized probes, *Q* are the quantification factors that indicate the amount of each expression pattern needed to

approximate the expression, *P* are the base expression unitary patterns that just depict the canonical shape patterns and *E* is the residual error from the approximation. The matrices are defined as:

$$\begin{bmatrix} S^{1*}_{Cy3} & \cdots & S^{n*}_{Cy3} \\ S^{1*}_{Cy5} & \cdots & S^{n*}_{Cy5} \end{bmatrix} \approx \begin{bmatrix} Q^{1}_{Cy3} & Q^{2}_{Cy3} \\ Q^{1}_{Cy5} & Q^{2}_{Cy5} \end{bmatrix} \bullet \begin{bmatrix} P^{1}_{1} & \cdots & P^{n}_{1} \\ P^{1}_{2} & \cdots & P^{n}_{2} \end{bmatrix}$$

Each *S* matrix component is a signal associated with a probe and a color, and the *Q* an *P* components are the result of the MCR algorithm.

As the MCR technique is a family of solutions, the following constraints were applied to solve the equation: all the matrices must be positive (transcripts can only be added, not subtracted), a wide range of intensities must be possible; only two events (the two colors) exist per gene, and a random noise due to all the different conditions. In practice, that translates into:

a) PCA filtering/whitening was used to minimize the impact of random noise.
b) The Alternate Least Squares (ALS) methodology using non-negative least squares approach was then used to find out the *Q* and *P* matrices from the initial expression matrix. To have an initial approach for the *Q* and *P* matrices and begin the iterative ALS, a principal component analysis (PCA) was used.
c) The expression vector was normalized to optimize the numerical stability for the iterative ALS method.
d) The two colors only allow defining n x 2 matrices, which can be deconvoluted in two components. The components do not necessarily have any biological meaning, i.e. they do not necessarily represent the actual transcripts of the mixture. However, the method is still valid to detect change, although the biological interpretation becomes much more difficult.

The complete flowchart for data analysis in AltPolyphemus (Additional file 2: Figure S9) was validated on data obtained from the hybridization of synthetic VEGF and PCBP4 transcripts on the pilot array. To eliminate false positives due to low expression probes, a yeast spike at very low concentration was used. This allowed monitoring and processing the genes with very low expression probes.

The presence of a bona fide splice event is expected to generate a variation in the Fold Change pattern. Whether this change can actually be detected depends on the quality of the probes, on their cross-hybridization level, on the magnitude of the change in transcript composition, on the number of alternative splicing events for the given gene that can give aggregate patterns, and on the general quality of the experiment. MCR-ALS was used to resolve the contribution of the sum of the different forms in the Cy3 and Cy5 channels. In the present study, a maximum number of 100 ALS iterations were run, applying the basic non-negative restrictions (which allowed optimization of algorithms such as fast non-negative linear squares, FNNLS). Since an iterative process can have several possible solutions, an optimization process using minimization of the maximum error component (E matrix) was defined. Finally, the resolved aggregated patterns were used to check the error in the modeling and to compare it to an expression model. An error from MCR-ALS less than Gene Expression indicated a possible differential splicing event. That translates to final gene expression pattern and quantification matrices having a rank of two and the residual error $E_{Isoform}$ being smaller than the error from single gene expression pattern approach E_{gene}.

$$E_{Isoform} \approx \begin{bmatrix} S^{1*}_{Cy3} & \cdots & S^{n*}_{Cy3} \\ S^{1*}_{Cy5} & \cdots & S^{n*}_{Cy5} \end{bmatrix} - \begin{bmatrix} Q^{1}_{Cy3} & Q^{2}_{Cy3} \\ Q^{1}_{Cy5} & Q^{2}_{Cy5} \end{bmatrix} \bullet \begin{bmatrix} P^{1}_{1} & \cdots & P^{n}_{1} \\ P^{1}_{2} & \cdots & P^{n}_{2} \end{bmatrix}$$

Isoform Hypothesis

$$E_{Expression} \approx \begin{bmatrix} S^{1*}_{Cy3} & \cdots & S^{n*}_{Cy3} \\ S^{1*}_{Cy5} & \cdots & S^{n*}_{Cy5} \end{bmatrix} - \begin{bmatrix} Q^{1}_{Cy3} & 0 \\ Q^{1}_{Cy5} & 0 \end{bmatrix} \bullet \begin{bmatrix} P^{1}_{1} & \cdots & P^{n}_{1} \\ 0 & \cdots & 0 \end{bmatrix}$$

Gene Expression Hypothesis

In analogy with the Fold Change obtained in standard gene-expression analyses, the arbitrary figure "Form Change" was empirically created to express the magnitude of the change in the expression ratio along the length of each transcript. The defined "Form Change" is as follows:

$$\mathrm{FormChange} = \log_{16}\left[\frac{\max\left(\frac{Q^{1}_{Cy3}}{Q^{1}_{Cy5}}, \frac{Q^{2}_{Cy3}}{Q^{2}_{Cy5}}\right)}{\min\left(\frac{Q^{1}_{Cy3}}{Q^{1}_{Cy5}}, \frac{Q^{2}_{Cy3}}{Q^{2}_{Cy5}}\right)}\right]$$

To allow for final visual interpretation, the algorithm was completed with a graphic interface that plots the Cy3 and Cy5 oligonucleotide intensities for all the oligonucleotides of a gene with signal intensity about background + 3σ of background variability, and with the resolved curves composing the signal in the Cy3 and Cy5 channels (Additional file 2: Figure S3).

As a summary, the implemented algorithm is able to detect and rank those genes that have a significant probability of showing an expression mixture with different transcripts contributions. Combined with the first-pass gene based analysis, the algorithm is able to detect the probes that are changing in the mixtures, allowing, together with the probe sequences and tagged probe quality, to make a biological hypothesis and plan the validation experiments.

Supplemental References

Anton MA, Gorostiaga D, Guruceaga E, Segura V, Carmona-Saez P, Pascual-Montano A, Pio R, Montuenga LM, Rubio A. **SPACE: an algorithm to predict and quantify alternatively spliced isoforms using microarrays**. *Genome Biol* 2008, **9**: R46.

Cerdà J, Mercadé J, Lozano JJ, Manchado M, Tingaud-Sequeira A, Astola A, Infante C, Halm S, Viñas J, Castellana B et al. **Genomic resources for a commercial flatfish, the Senegalese sole (Solea senegalensis): EST sequencing, oligo microarray design, and development of the Soleamold bioinformatic platform.** *BMC Genomics* 2008, **9**:508.

de Juan, A. and Tauler, R. **Chemometrics applied to unravel multicomponent processes and mixtures: Revisiting latest trends in multivariate resolution**. *Analytica Chimica Acta* 2003, **500**: 195-210.

de Juan, A. and Tauler, R. **Comparison of three-way resolution methods for non-trilinear chemical data sets**. *Journal of Chemometrics* 2001, **15**: 749-771.

Dudoit, S., Yang, Y.H., Callow, M.J., and Speed, T.P. **Statistical methods for identifying differentially expressed genes in replicated cDNA microarray experiments**. *Statistica Sinica* 2002, **12**: 111-139.

Jaumot J, Tauler R and Gargallo R. **Exploratory data analysis of DNA microarrays by multivariate curve resolution**. *Analytical Biochemistry* 2006, **358**: 76-89.

Tauler, R. **Multivariate curve resolution applied to second order data. Chemometrics and Intelligent Laboratory Systems** 1995, **30**: 133-146.

Tauler, R., Smilde, A., and Kowalski, B.R. **Selectivity, local rank, three-way data analysis and ambiguity in multivariate curve resolution**. *Journal of Chemometrics* 1995, **9**: 31-58.

Wang H, Hubbell E, Hu JS, Mei G, Cline M, Lu G, Clark T, Siani-Rose MA, Ares M, Kulp DC et al. **Gene structure-based splice variant deconvolution using a microarray platform**. *Bioinformatics* 2003, **19 Suppl 1**: i315-322.

Wentzell PD, Karakach TK, Roy S, Martinez MJ, Allen CP and Werner-Washburne M. **Multivariate curve resolution of time course microarray data**. BMC Bioinformatics 2006, **7**: 343.

Workman C, Jensen LJ, Jarmer H, Berka R, Gautier L, Nielser HB, Saxild HH, Nielsen C, Brunak S, and Knudsen S. **A new non-linear normalization method for reducing variability in DNA microarray experiments**. *Genome Biol* 2002, **3**: research0048.

Supplementary Tables

Table S1. Lung cancer patients of the array set.

No.	Lung cancer subtype	Category	Stage	TNM	Gender	Age	Smoker
51	Squamous carcinoma	Poorly differentiated	IB	T2N0	F	63	Yes
NM21	Squamous carcinoma	Poorly differentiated	IA	T1N0	M	60	Yes
133	Squamous carcinoma	Moderately differentiated	IB	T2N0	M	63	Yes
V56	Squamous carcinoma	Poorly differentiated	IB	T2N0	M	67	Yes
V57	Squamous carcinoma	Poorly differentiated	IB	T2N0	M	78	NA
V60	Squamous carcinoma	Moderately differentiated	IA	T1N0	M	81	Yes
V71	Adenocarcinoma	Poorly differentiated	IB	T2N0	M	74	NA
V72	Adenocarcinoma	Poorly differentiated	IIB	T2N1	M	41	NA
V78	Adenocarcinoma	Well differentiated	IA	T1N0	M	75	Yes
V79	Adenocarcinoma	Moderately differentiated	IB	T2N0	M	74	NA
V83	Adenocarcinoma	Moderately differentiated	IIB	T2N1	M	73	Yes
V89	Adenocarcinoma	Poorly differentiated	IIIA	T3N2	M	74	Yes
V92	Adenocarcinoma	Poorly differentiated	IIB	T2N1	M	76	Yes
V95	Adenocarcinoma	Well differentiated	IB	T2N0	M	80	Yes
V107	Squamous carcinoma	Moderately differentiated	IIB	T3N0	F	83	NA
V115	Squamous carcinoma	Poorly differentiated	IA	T1N0	M	70	Yes
V118	Squamous carcinoma	Moderately differentiated	IA	T1N0	M	46	Yes
V113	Squamous carcinoma	Poorly differentiated	IIA	T1N1	M	45	Yes
V61	Squamous carcinoma	Poorly differentiated	IB	T2N0	M	55	Yes
113	Squamous adenoma	NA	IB	T2N0	M	56	Yes

NA: data not available.

Table S2. Lung cancer patients of the series for validation.

No.	Lung cancer subtype	Category	Stage	TNM	Sex	Age	Smoker
50	Squamous carcinoma	Moderately differentiated	IB	T2N0	M	50	Yes
60	Adenocarcinoma	Poorly differentiated	IB	T2N0	F	52	No
63	Squamous carcinoma	Moderately differentiated	IA	T1N0	M	75	Yes
80	Adenocarcinoma	Well differentiated	IIIA	T2N2	M	52	Yes
86	Adenocarcinoma	NA	IIB	T2N1	F	73	No
102	Squamous carcinoma	Poorly differentiated	IA	T1N0	M	55	Yes
111	Squamous carcinoma	Poorly differentiated	IB	T2N0	M	45	Yes
118	Adenocarcinoma	Moderately differentiated	IB	T2N0	F	78	No
122	Adenocarcinoma	Poorly differentiated	IB	T2N0	M	51	Yes
126	Large cell carcinoma	NA	IIB	T3N0	M	68	Yes
V14	Squamous carcinoma	Moderately differentiated	IB	T2N0	M	54	Yes
V20	Squamous carcinoma	Poorly differentiated	IIB	T2N1	M	62	Yes
V30	Squamous carcinoma	Poorly differentiated	IB	T2N0	M	63	Yes
V33	Squamous carcinoma	Well differentiated	IB	T2N0	M	57	Yes
V40	Squamous carcinoma	Poorly differentiated	IIB	T2N1	M	69	No
V43	Squamous carcinoma	Moderately differentiated	IIA	T2N1	M	49	Yes
V48	Squamous carcinoma	Poorly differentiated	IIB	T2N1	M	78	Yes
V53	Squamous carcinoma	Poorly differentiated	IB	T2N0	M	67	Yes
V88	Adenocarcinoma	Poorly differentiated	IIIA	T2N2	M	75	Yes
V128	Squamous carcinoma	Poorly differentiated	IIIA	T3N1	M	75	NA
V141	Squamous carcinoma	Poorly differentiated	IIB	T2N1	M	63	Yes
V144	Squamous carcinoma	Poorly differentiated	IIB	T2N1	M	77	Yes

Table S3. Primers used for PCR.

Gene	Primer	Sequence (5´- 3´)
CEACAM1	01	CCACTTCACAGAGTGCGTGT
	06	TGGACAGTTCCATGTATACCATA A
	08	CTTGTGGTAGAGCATTATGG
	09	TAGGTGGGTCATTGGAGTGG
	14	AGCCTGGGTAACATGGTGAG
	15	TCTTGTGGTAGAGCATTATCAGT
	16	ATACCTGCCACGCCAATAAC
	17	TCTCAGCACTTTGGGAAACA
	18	CAAGCAATCCTCCCATCTG
	20	AAGACGATCATAGTCACTGATAA
FHL1	01	AGAACCCCATCACTGGGTTTG
	02	GCATTTTTTGCAGTGGAAGCA
	03	GAACCCCATCACTGGGAAAA
	04	CGTTTCCCGTGGCACACT
MLPH	02	AGGGCCTCCTCCTCTACATC
	03	GGGCGTCTTCTGAGAGTCA
SUSD2	05	CGCTCGTCTATGTGCTGCT
	08	GACAGAGTAGGGGGTTAAAT
	10	CCAAACTATGTGGGGACGAT
	12	CTGCTGCCTGAGAAGTTCCT
	13	AGTTGTGGACCAGGAACCAG

	14	AGGTACCTGTTGCCCTCCTT
	15	CAAAAGGAGGGCAACAGGTA
	16	AAGATGATGCCCAACAGCAC

Table S4. Cancer-associated splice variants in lung cancer cell lines determined by conventional RT-PCR.

		CEACAM1			***SUSD2***			
**Cell Line**	**Histology** *	1-1	1-3	1-3A	Intron11	Intron12	Intron13	Intron14
H69	SCLC	+	-	-	-	-	+	+
H82	SCLC	-	-	-	+	+	+	+
H187	SCLC	+	-	-	-	+	+	+
H209	SCLC	-	-	-	-	-	+	+
H23	AC	+	-	-	-	-	+	+
H441	AC	+	-	-	-	+	+	+
H1648	AC	+	-	-	-	-	-	+
H2087	AC	+	-	-	-	-	-	+
HCC44	AC	-	-	-	-	-	+	-
HCC827	AC	+	-	+	+	+	-	+
A549	BAC	-	-	-	-	-	-	+
H322	BAC	-	-	-	-	+	+	+
H358	BAC	+	+	+	-	-	-	-
H1385	SCC	+	-	-	-	+	+	+
HTB-58	SCC	-	-	+	-	-	-	-
H157	SCC	-	-	-	-	-	+	+
H226	SCC	+	-	-	-	-	-	-
272H	SCC	+	-	+	-	+	+	+
HCC15	SCC	+	+	-	-	+	+	+

H460	LCC	+	-	-	-	+	+	-
H1299	LCC	-	-	+	-	+	+	+
H661	LCC	+	-	-	-	-	+	+
H720	Carcinoid	+	+	+	+	+	+	+

* SCLC: small cell lung carcinoma; AC: adenocarcinoma; BAC: bronchioloalveolar carcinoma; SCC: squamous cell carcinoma; LCC: large cell carcinoma.
